# The *Physcomitrella patens* unique alpha-dioxygenase participates in both developmental processes and defense responses

**DOI:** 10.1186/s12870-015-0439-z

**Published:** 2015-02-12

**Authors:** Lucina Machado, Alexandra Castro, Mats Hamberg, Gerard Bannenberg, Carina Gaggero, Carmen Castresana, Inés Ponce de León

**Affiliations:** Departamento de Biología Molecular, Instituto de Investigaciones Biológicas Clemente Estable, Avenida Italia 3318, CP 11600 Montevideo, Uruguay; Department of Medical Biochemistry and Biophysics, Division of Physiological Chemistry II, Karolinska Institutet, 17177 Stockholm, Sweden; Departamento de Genética Molecular de Plantas, Centro Nacional de Biotecnología, Consejo Superior de Investigaciones Científicas, 28049 Madrid, Spain; Laboratorio de Biología Molecular Vegetal, Facultad de Ciencias, Universidad de la República, Iguá 4225, CP 11400 Montevideo, Uruguay

**Keywords:** α-dioxygenases, *Physcomitrella patens*, Development, Defense, *Pectobacterium*, *Botrytis cinerea*

## Abstract

**Background:**

Plant α-dioxygenases catalyze the incorporation of molecular oxygen into polyunsaturated fatty acids leading to the formation of oxylipins. In flowering plants, two main groups of α-DOXs have been described. While the α-DOX1 isoforms are mainly involved in defense responses against microbial infection and herbivores, the α-DOX2 isoforms are mostly related to development. To gain insight into the roles played by these enzymes during land plant evolution, we performed biochemical, genetic and molecular analyses to examine the function of the single copy moss *Physcomitrella patens* α-DOX (Ppα-DOX) in development and defense against pathogens.

**Results:**

Recombinant Ppα-DOX protein catalyzed the conversion of fatty acids into 2-hydroperoxy derivatives with a substrate preference for α-linolenic, linoleic and palmitic acids. Ppα-DOX is expressed during development in tips of young protonemal filaments with maximum expression levels in mitotically active undifferentiated apical cells. In leafy gametophores, Ppα-DOX is expressed in auxin producing tissues, including rhizoid and axillary hairs. Ppα-DOX transcript levels and Ppα-DOX activity increased in moss tissues infected with *Botrytis cinerea* or treated with *Pectobacterium carotovorum* elicitors. In *B. cinerea* infected leaves, Ppα-DOX-GUS proteins accumulated in cells surrounding infected cells, suggesting a protective mechanism. Targeted disruption of Ppα-DOX did not cause a visible developmental alteration and did not compromise the defense response. However, overexpressing Ppα-DOX, or incubating wild-type tissues with Ppα-DOX-derived oxylipins, principally the aldehyde heptadecatrienal, resulted in smaller moss colonies with less protonemal tissues, due to a reduction of caulonemal filament growth and a reduction of chloronemal cell size compared with normal tissues. In addition, Ppα-DOX overexpression and treatments with Ppα-DOX-derived oxylipins reduced cellular damage caused by elicitors of *P. carotovorum*.

**Conclusions:**

Our study shows that the unique α-DOX of the primitive land plant *P. patens*, although apparently not crucial, participates both in development and in the defense response against pathogens, suggesting that α-DOXs from flowering plants could have originated by duplication and successive functional diversification after the divergence from bryophytes.

**Electronic supplementary material:**

The online version of this article (doi:10.1186/s12870-015-0439-z) contains supplementary material, which is available to authorized users.

## Background

Oxylipins are a diverse group of oxygenated fatty acids which are involved in controlling plant development and defense against microbial pathogens and insects [1,2]. The biosynthesis of oxylipins is catalyzed by fatty acid oxygenases including lipoxygenases (LOXs) and α-dioxygenases (α-DOXs), which add molecular oxygen to polyunsaturated fatty acids, mainly linolenic (18:3) and linoleic (18:2) acids leading to hydroperoxide formation [3,4]. While LOXs are located in chloroplasts [3], α-DOXs are found in oil bodies and endoplasmic reticulum-like structures [5]. LOXs catalyze the incorporation of molecular oxygen into these fatty acids at either carbon positions 9 or 13, leading to 9- and 13-hydroperoxy fatty acids, which are further metabolized to various lipid mediators including jasmonates and volatile aldehydes [3,6]. LOX-derived oxylipins have important functions in a variety of plant processes such as seed development, germination, vegetative growth, lateral root development and in defense responses against wounding, insect feeding and microbial infection [1,2,7-10]. α-DOXs add molecular oxygen to the α-carbon (C-2) of a broad range of fatty acids leading to the formation of chemically unstable 2(R)-hydroperoxy fatty acids which are either reduced to 2(R)-hydroxy fatty acid or spontaneously decarboxylated to the corresponding shorter chain fatty aldehyde [4,11,12]. Two main groups of α-DOXs have been described in flowering plants. The α-DOX1 type enzymes are mainly involved in defense responses against microbial infection and herbivores, while the α-DOX2 type enzymes are more related to development. α-DOX1 transcripts accumulate rapidly in tobacco, *Arabidopsis thaliana* and *Capsicum annuum* leaves after pathogen assault [11,13-15]. *A. thaliana* plants with low or null α-DOX1 activity are more susceptible to *Pseudomonas syringae*, as evidenced by increased bacterial growth and symptom development in inoculated leaves, suggesting a possible role in protecting plant tissues against oxidative stress and cell death generated by pathogens [13,16]. In addition, Arabidopsis *α-dox1* mutants showed an impaired systemic response against *P. syringae* in distal leaves [16]. In *Nicotiana attenuata α-DOX1* transcripts are weakly induced by pathogen infection, while *Naα-DOX1* is highly expressed by herbivore attack and plays an important role in the anti-herbivore defense response of this plant [17,18]. In tomato and *A. thaliana* α-DOX1 is also needed for basal resistance against aphids [19]. The second isoform, α-DOX2, is expressed in *A. thaliana* seedlings, during senescence induced by detachment of *A. thaliana* leaves and in flowers, while it is not induced after pathogen inoculation [20,21]. *Naα-DOX2* is expressed in senescent leaves, in flowers and roots but not in seedlings [17]. *Solanum lycopersicum* knockout mutants of α-DOX2 and *N. attenuata* co-silenced α-DOX1 and α-DOX2 plants have a stunted phenotype [17,22]. The latter result suggests that Naα-DOX1 can also regulate development and has distinct and overlapping function with Naα-DOX2 [17]. Complementation of tomato α-DOX2 mutant with Atα-DOX2 partially restores the compromised growth phenotype [21]. However, *A. thaliana* α-DOX2 mutant did not have an altered developmental phenotype [21], suggesting that the role played by α-DOX2 in development is species specific.

The moss *Physcomitrella patens* is an excellent plant model species to perform functional studies of individual genes by reverse genetics, due to its high rate of homologous recombination, comparable to yeast cells, that enables targeted gene disruption [23]. In addition, given its phylogenetic position as an early diverging land plant between green algae and flowering plants, it represents an interesting model plant to perform evolutionary studies of the role played by genes in developmental and defense processes. *P. patens* is infected by several known plant pathogens, including *Pectobacterium* species, *Botrytis cinerea*, and *Pythium* species, and in response to infection defense mechanisms similar to those induced in flowering plants are activated [24-26]. Recently, several studies have shown that in *P. patens* the LOX pathway is similar to that of flowering plants but it presents some unique features. In addition to 18:3 and 18:2 unsaturated C18 fatty acids, C20 fatty acids, which are absent in flowering plants, are substrates for *P. patens* LOXs leading to the formation of a structurally diverse group of oxylipins [27-29]. While *P. patens* accumulates the precursor of jasmonic acid, 12-oxophytodienoic acid (OPDA), in response to pathogen infection or wounding [26,30], no jasmonic acid has been detected, suggesting that only the plastid-localized part of the LOX pathway is present in this moss [30]. *P. patens* has only one gene encoding a putative α-DOX (Ppα-DOX); this showed 49–53% identity to α-DOXs of flowering plants and possessed the two conserved heme-binding histidines [20]*.* Ppα-DOX activity was detected in homogenized tissues of *P. patens* leading to the generation of 2-hydroxypalmitic acid [20]*.* The expression of the putative Ppα-DOX in a baculovirus system further showed that this enzyme is capable of oxygenating 3-oxalinolenic acid similarly to Atα-DOX1 leading to the production of the same oxylipins [31]. In this study, we have analyzed Ppα-DOX function in more detail. We showed that Ppα-DOX is highly expressed in mitotically active apical cells of protonemal filaments and rhizoids, in auxin-producing cells of gametophores, and in pathogen-infected and elicitor-treated tissues. Ppα-DOX knockout mutants did not have a visible developmental alteration and were not compromised in the defense response. However, overexpressing Ppα-DOX, or treating wild-type plants with Ppα-DOX-derived oxylipins, altered moss development and led to reduced cellular damage caused by *P. carotovorum* elicitors.

## Results

### *P. patens* α-Dioxygenase activity

**Figure 1:**
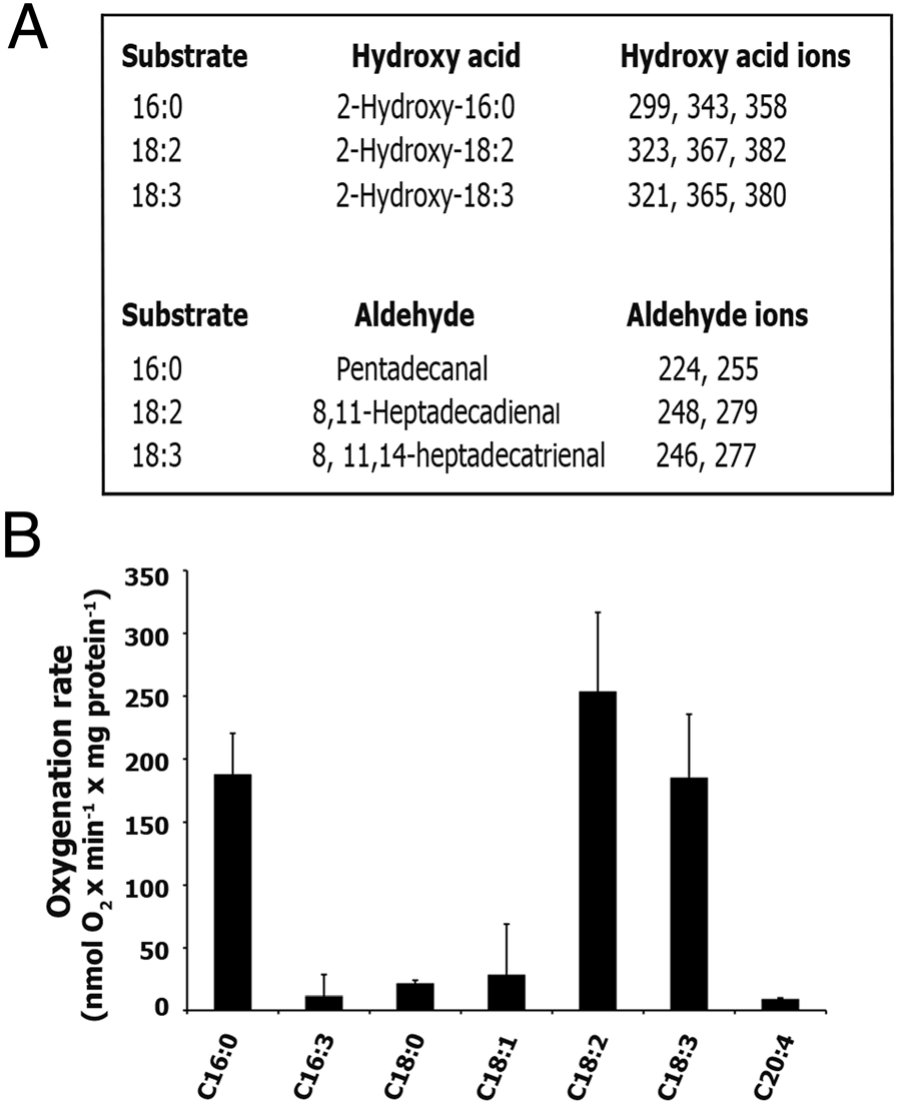
**Determination of α-dioxygenase activity of Ppα-DOX. (A)** Mass-spectral ions (*m/z*) recorded on Ppα-DOX-derived 2-hydroxy fatty acids (methyl ester/trimethylsilyl ether derivatives) and fatty aldehydes (O-methyloxime derivatives). **(B)** Fatty acid substrate specificity of oxygenation by Ppα-DOX (mean +/− SE of n = 3–5 measurements).

In previous work, we obtained High Five insect cells containing recombinant Ppα-DOX-expressing baculovirus [31] and the products formed by α-oxygenation of 16:0 were determined [20]. Here, homogenates of these insect cells were incubated with different fatty acids, leading to the generation of 2-hydroperoxides as shown by the identification of the corresponding aldehydes and 2-hydroxy acids (Figure 1A, Additional file 1). The fatty acid substrate specificity of Ppα-DOX was determined by oxygen consumption assays and the results showed that palmitic (16:0), linoleic (18:2) and linolenic acid (18:3) were the most efficiently oxygenated substrates (Figure 1B). Under our experimental conditions, Ppα-DOX was not capable of using arachidonic acid (20:4) as a substrate. No α-DOX activity was detected when homogenates from High Five insect cells infected with baculovirus prepared from empty pFastBac vector were incubated with different fatty acids. These results confirm that Ppα-DOX is an α-dioxygenase, and demonstrate that it can oxygenate fatty acids with preferences for palmitic (16:0), linoleic (18:2) and linolenic acid (18:3). The products obtained (Figure 1A), i.e. the 2-hydroxy derivatives of 16:0, 18:2 and 18:3 and the aldehydes pentadecanal, 8,11-heptadecadienal and 8,11,14-heptadecatrienal were identified by GC-MS analysis as previously described [4,15,20]. Further support for the formation of pentadecanal and 2-hydroxy-16:0 from 16:0 was provided by GC-MS analyses run in the selected-ion-monitoring mode using synthetically prepared trideuterated standards (Additional file 2).

### Phylogenetic relationship of Ppα-DOX and other plant α-Dioxygenases

**Figure 2:**
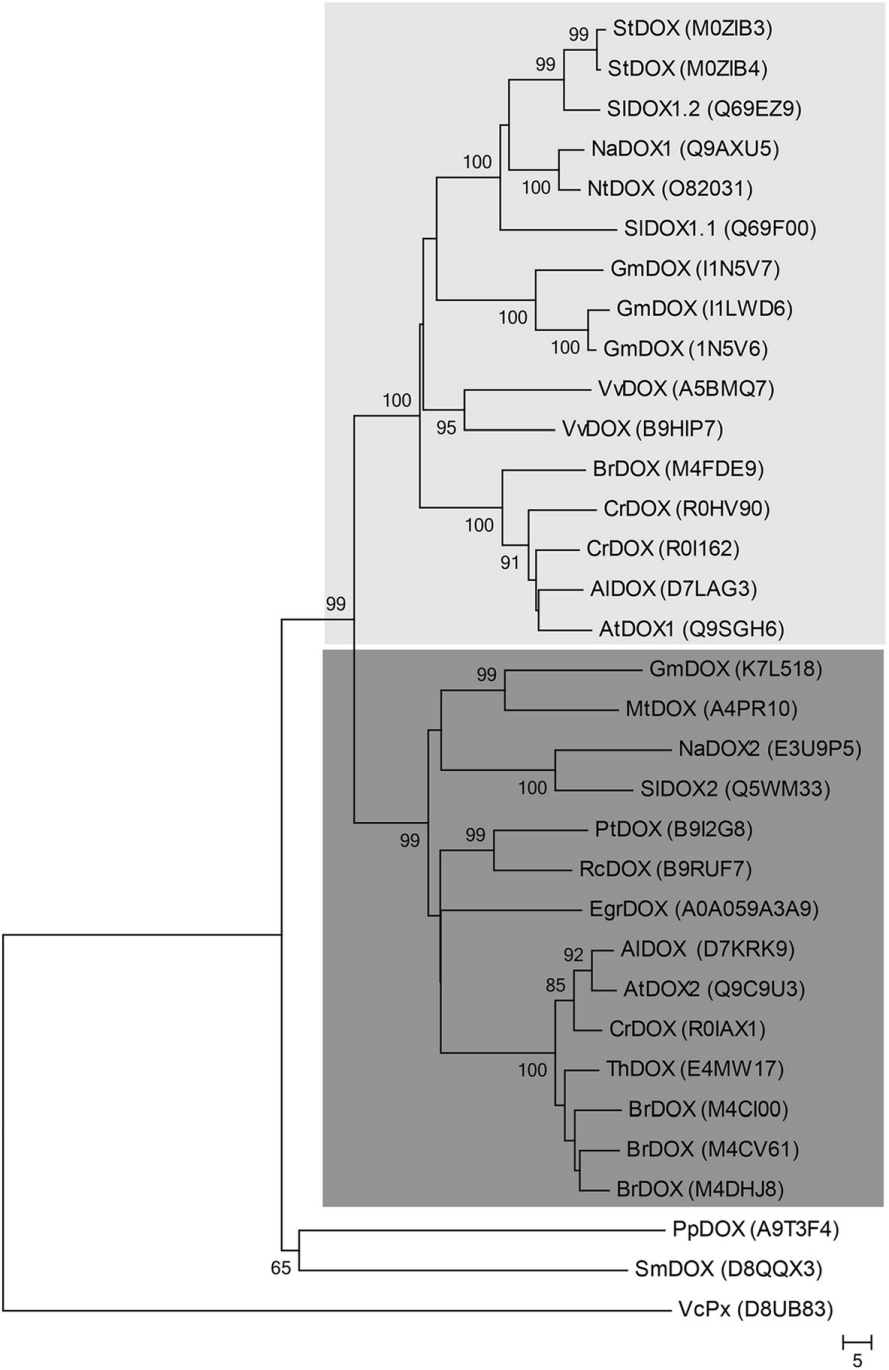
**Phylogenetic tree of confirmed and putative α-dioxygenases.** Full-length amino acid sequences of available and putative α-DOX proteins were aligned by CLUSTAL W and a phylogenetic tree was constructed by the neighbor-joining method using MEGA version 5.05. Numbers at branch nodes represent the confidence level of 1000 bootstrap replications. The identities of the individual α-DOX protein sequences are indicated by their uniprot entry number (http://www.uniprot.org). Two clusters are highlighted in the phylogenetic tree including α-DOX1 (light grey) and α-DOX2 proteins (dark gray) respectively. The abbreviations of species are as follows: Al: *Arabidopsis lyrata*, At: *Arabidopsis thaliana*, Br: *Brassica rapa*, Cr: *Capsella rubella*, Eg: *Eucalyptus grandii*, Gm: *Glycine max*, Mt: *Medicago truncatula*, Na: *Nicotiana attenuata*, Nt: *Nicotiana tabacum*, Pp: *Physcomitrella patens*, Pt: *Populus trichocarpa*, Rc: *Ricinus communis*, Sl: *Solanum lycopersicum*, Sm: *Selaginella moellendorffii*, St: *Solanum tuberosum*, Th: *Thellungiella halophila*, Vc: *Volvox carteri*, Vv: *Vitis vinifera*.

Expanding previous phylogenetic analyses of plant α-DOXs [17,20,21], a phylogenetic tree was constructed with Ppα-DOX and other confirmed and putative α-DOXs, including a putative algae α-DOX. Full-length amino acid sequences were aligned with CLUSTAL W, and a phylogenetic tree was constructed by the Neighbor joining method using MEGA version 5.05 software (Figure 2). The tree shows four clear clusters, one represented by α-DOX1 type enzymes and another by α-DOX2 type enzymes from flowering plants. Ppα-DOX and its lycophyte α-DOX homologue (*Selaginella moellendorffii*, which belongs to a primitive group of vascular plants), form a clearly separated cluster from α-DOXs of flowering plants at the base of the plant clade. The putative α-DOX of the multicellular green algae *Volvox carteri* is placed in the fourth separate cluster.

### Ppα-DOX-GUS accumulation patterns during gametophyte development

**Figure 3:**
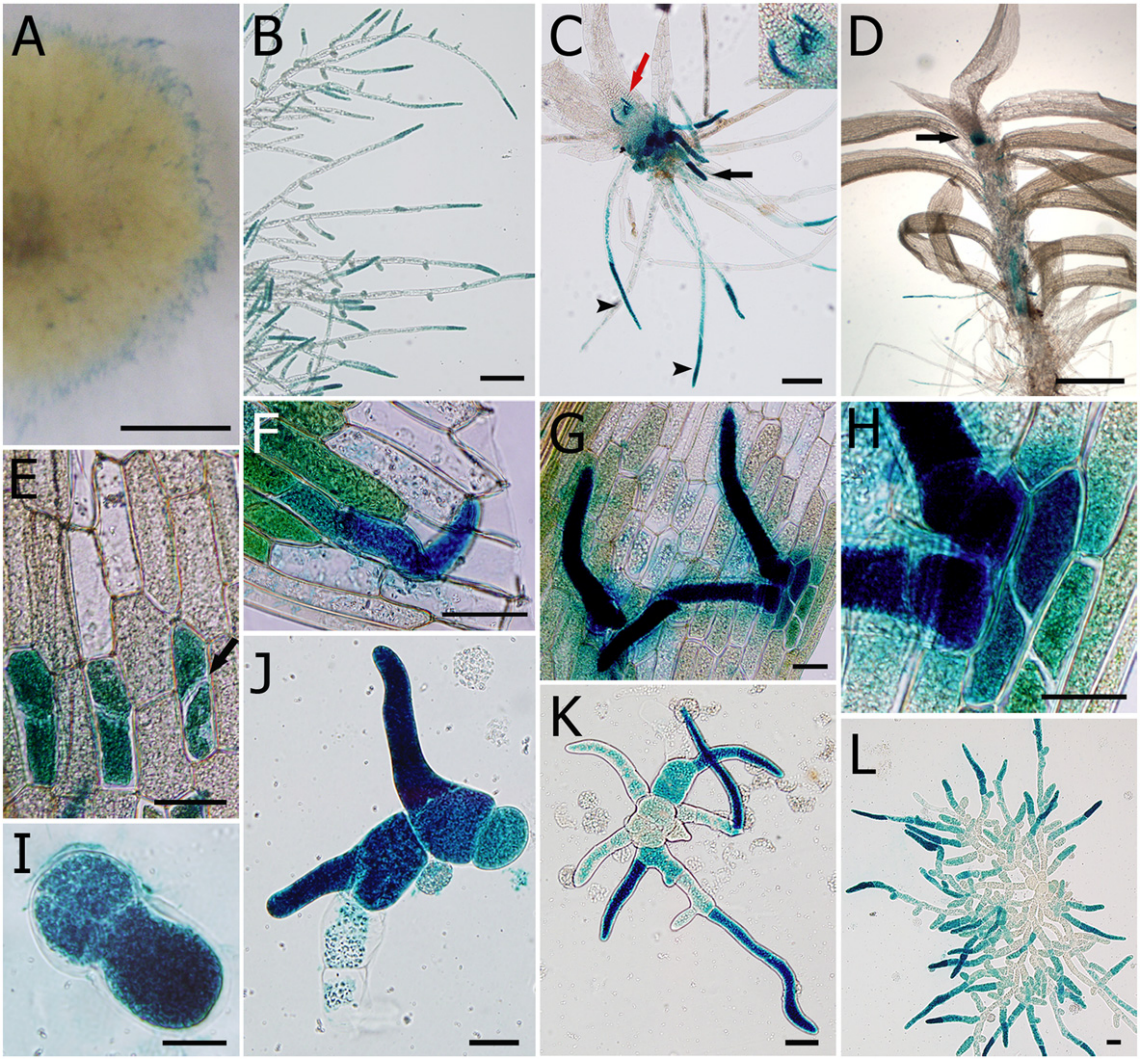
**Ppα** **-DOX expression in** ***P. patens*** **tissues.** GUS staining of Ppα-DOX-GUS lines in; **(A)** border of a colony, **(B)** protonemal tissues at the border of a colony, **(C)** juvenile gametophore with GUS-stained young rhizoids (black arrow), GUS-stained long rhizoids with maximum staining in apical cells (arrowheads) and GUS-stained axillary hairs (red arrow), inset shows magnified axillary hairs, **(D)** adult gametophore with GUS-stained cells in the shoot apex (arrow) and parts of the cauloid, **(E)** GUS-stained dividing cells in detached leaf showing septa of cells that divided after leaf detachment (arrow), **(F)** protruded chloronemal cell facing the cut of the leaf, **(G)** protruded chloronemal cells of a detached leaf, **(H)** a closer view of G, **(I)** protoplast after regeneration for 4 days, **(J)** protoplasts after regeneration for 6 days, **(K)** protoplasts after regeneration for 7 days, and **(L)** young moss colony after protoplast regeneration for 11 days. Scale bars:20 μm in **E-L**; 100 μm in B; 0,5 mm **C**, **D**; 0,5 cm in **A**.

To investigate the spatiotemporal expression patterns of the *Ppα-DOX* gene in *P. patens* tissues, the reporter *uidA* gene encoding β-glucuronidase (GUS), was inserted in frame just before the stop codon of the *Ppα-DOX* gene by means of homologous recombination. After transformation, two stable Ppα-DOX-GUS lines, Ppα-DOX-GUS-12 and Ppα-DOX-GUS-2, expressing the corresponding fusion proteins from the native Ppα-DOX promoter were selected for further studies (Additional file 3). Haploidy of both lines was confirmed by measuring nuclear DNA content (data not shown). Results from PCR-based genotyping and Southern blot analysis showed that Ppα-DOX-GUS-12 has incorporated one single copy of the construct by two events of homologous recombination in the Ppα-DOX locus, while Ppα-DOX-GUS-2 showed an integration event at only one border and had more than one insertion in its genome (Additional file 3). Ppα-DOX-GUS-2 α-DOX activity was similar as wild-type plants, while Ppα-DOX-GUS-12 did not show α-DOX activity. Differences in integration events leading to differences in protein folding could explain the lack or presence of Ppα-DOX activity in these lines. To discard the presence of a wild-type copy of Ppα-DOX adjacent to the inserted construct in Ppα-DOX-GUS-2, PCR amplification was performed using primers DOX3F+3′DOXr. The corresponding fragment of 2209 pb was only amplified in the WT, indicating the absence of a full wild-type copy in the transformants (Additional file 3). Both reporter lines revealed identical overall GUS staining patterns, suggesting that α-DOX derived oxylipins do not affect Ppα-DOX-GUS expression (Additional file 3). Ppα-DOX-GUS protein accumulation pattern in the juvenile gametophyte phase revealed their presence in tips of protonemal filaments growing at the edge of the colonies (Figure 3A). The highest Ppα-DOX-GUS accumulation occurs in protonemal apical cells, gradually diminishes in the adjacent differentiated subapical cells, and is absent in the remaining older proximate protonemal cells (Figure 3B). Leafy gametophores showed Ppα-DOX expression in short and long rhizoids with maximum expression levels in apical cells of rhizoids (Figure 3C), and in axillary hairs and the shoot apex (Figure 3C and D). Ppα-DOX-GUS accumulation was also observed in some parts of the cauloid while no visible staining was detectable in leaves (Figure 3D). This expression pattern correlates with sites of auxin synthesis and auxin response in gametophores [32-34]. We therefore decided to evaluate auxin responsiveness of the Ppα-DOX promoter in moss colonies grown in the presence of 5 μM NAA for 2 days. Ppα-DOX-GUS expression was clearly enhanced after NAA treatment in cauloids and leaves of gametophores (Additional file 4). Apical cells of protonemal filaments and rhizoids are mitotically active cells, with characteristics of stem cells [35-37], suggesting that Ppα-DOX expression is enhanced in these type of cells. We therefore analyzed Ppα-DOX-GUS expression in other *P. patens* stem cells, including cells of detached gametophore leaves which divide and give rise to chloronemal apical stem cells [38], and apical cells from regenerating protoplasts [39]. The results show that Ppα-DOX is expressed in cells that divide after leaf detachment (Figure 3E), and in chloronemal apical stem cells from dissected leaves which start to protrude with tip growth (Figure 3F-H). In addition, Ppα-DOX-GUS expression was detected in regenerating protoplasts, which start tip growth by dividing asymmetrically with the maximum expression levels in apical cells (Figure 3I-L). After several days of protoplasts regeneration, Ppα-DOX-GUS accumulation disappeared in the central nondividing protonemal cells. Taken together, the results indicate that Ppα-DOX is highly expressed in mitotically active cells, in auxin producing sites of gametophores, and in cauloids and leaves of auxin-treated plants.

### Pp*a*-DOX is induced after pathogen infection and elicitor treatment

**Figure 4:**
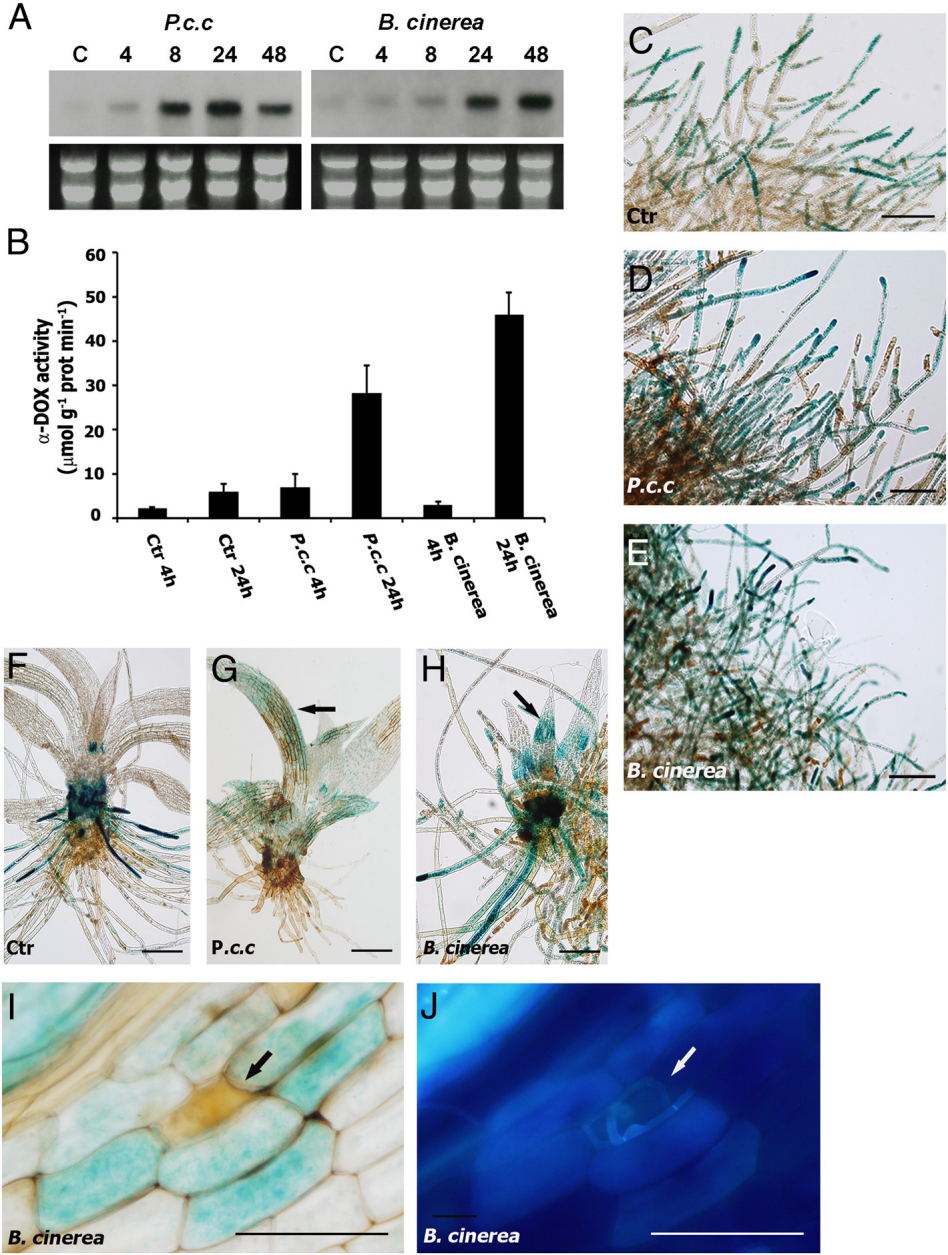
**Ppα-DOX expression and Ppα-DOX activity in response to** ***Pectobacterium*** **elicitors and spores of** ***B. cinerea*** **. (A)** Expression of *Ppα-DOX* in response to elicitors of *P.c. carotovorum* (*P.c.c*) and spores of *B. cinerea* at different hours after treatments. **(B)** Ppα-DOX activity in tissues treated with water (Ctr), elicitors of *P.c. carotovorum* (*P.c.c*), and spores of *B. cinerea* at 4 and 24 hours. GUS accumulation in protonemal tissues of Ppα-DOX-GUS-2 line treated for 24 hours with water **(C)**, elicitors of *P.c. carotovorum* **(D)**, and spores of *B. cinerea* **(E)**. GUS accumulation in gametophores of Ppα-DOX-GUS-2 treated for 24 hours with water **(F)**, elicitors of *P.c. carotovorum* **(G)**, and spores of *B. cinerea* **(H)**. GUS accumulation in leaves treated with elicitors of *P.c. carotovorum* or spores of *B. cinerea* are indicated with an arrow. **(I)** GUS accumulation in a *B. cinerea*-infected leaf showing Ppα-DOX expression in cells surrounding a cell, which is infected with *B. cinerea* as evidenced by hyphae staining with the fluorescent dye solophenyl flavine 7GFE 500 **(J)**. Scale bars: 100 μm in C-E; 300 μm in F-H; 20 μm in I-J. A brown infected cell in I, and hyphae in J are indicated with a black and white arrow respectively.

Since α-DOX1 expression is induced after pathogen infection in flowering plants [11,13], *Ppα-DOX* transcript levels were evaluated in moss tissues in response to treatment with elicitors of *Pectobacterium carotovorum subsp. carotovorum* (*P.c. carotovorum*) (ex *Erwinia carotovora* subsp. *carotovora*), and inoculation with spores of *Botrytis cinerea* (*B. cinerea*). *Ppα-DOX* expression increased significantly after 8 hours with *P.c. carotovorum* elicitor treatment and after 24 hours with *B. cinerea* inoculation (Figure 4A), which correlates with an increase of fungal biomass [26]. Ppα-DOX activity increased significantly after 24 hours treatment with *P.c. carotovorum* elicitors and *B. cinerea* spores suspension (Figure 4B). In the Ppα-DOX-GUS-2 reporter line Ppα-DOX expression increased in protonemal tissues and leaves treated with elicitors of *P.c. carotovorum* or infected with *B. cinerea*, compared to control tissues (Figure 4C-H). Ppα-DOX-GUS-12 revealed identical GUS staining patterns (data not shown). Semi-quantitative RT-PCR confirmed the presence of Ppα-DOX-GUS fused transcripts only in Ppα-DOX-GUS-12, probably due to the multiple integration events in Ppα-DOX-GUS-2. In Ppα-DOX-GUS-12, levels of the fused Ppα-DOX-GUS transcript increased in elicitors-treated tissues compared to water-treated tissues (Additional file 3). When *B. cinerea*-inoculated leaves were analyzed in more detail, GUS expression was detected in cells surrounding *B. cinerea*-infected cells (Figure 4I). Most of these Ppα-DOX expressing cells also showed staining with the fluorescent dye solophenyl flavine 7GFE 500 (Figure 4J), suggesting changes in the cell walls which could be indicative of cell wall reinforcement [26]. Thus, Ppα-DOX expression and activity increased after *B. cinerea* infection and *P.c. carotovorum* elicitor treatments. In addition, Ppα-DOX is expressed in leaf cells surrounding *B. cinerea*-infected cells.

### Effects of α-DOX-derived oxylipins on moss development

**Figure 5:**
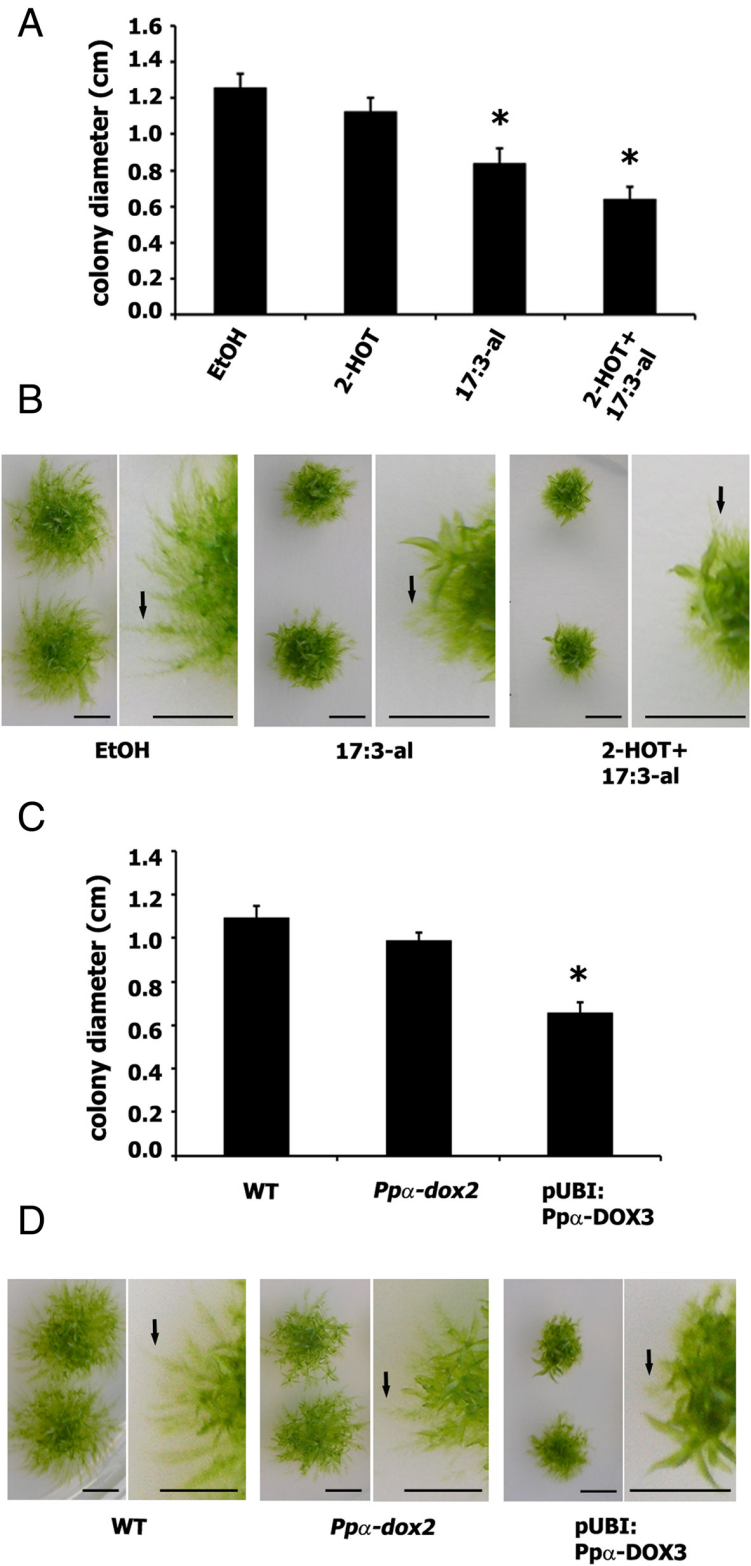
**Effect of α-DOX-derived oxylipins on moss colony morphology. (A)** Size of single moss colonies grown for 21 days in 50 μM of 2-HOT, 50 μM of 17:3-al or 50 μM 2-HOT+ 50 μM 17:3-al containing BCDAT medium, measured as diameter in centimeters, relative to control moss colonies grown on 0.5% ethanol. **(B)** Representative individual colonies and closer views showing the typical phenotype after 21 days of growth on 50 μM 17:3-al or 50 μM 2-HOT+ 50 μM 17:3-al-containing medium in comparison with control plants grown on 0.5% ethanol. **(C)** Size of wild-type (WT), *Ppα-dox-2* and pUBI:Ppα-DOX-3 moss colonies grown for 21 days in BCDAT medium measured as diameter in centimeters. **(D)** Representative individual colonies and closer views of WT, *Ppα-dox-2* and pUBI: Ppα-DOX3 showing the typical phenotype after 21 days of growth. Arrows in B and D indicate protonemal filaments. Results and standard deviation correspond to 16 colonies per sample. Asterisks for colonies grown on 50 μM of 17:3-al or 50 μM 2-HOT+ 50 μM 17:3-al, or pUBI:Ppα-DOX-3 colonies, indicate that the values are significantly different from control plants, or WT plants, respectively, according to Kruskal–Wallis test: P <0.001. Arrows indicate representative protruding caulonemal filaments. Scale bars represent 0,5 cm.

Since Ppα-DOX is highly expressed in apical protonemal cells which divide and give rise to typical moss colonies, we examined whether α-DOX-derived oxylipins could alter colony morphology. Small pieces of protonemal tissue of 1 mm were applied on medium containing 50 μM of α-DOX products derived from linolenic acid (18:3), including 2(R)-Hydroxy-9(Z),12(Z),15(Z)-octadecatrienoic acid (2-HOT), 8(Z),11(Z),14(Z)-heptadecatrienal (17:3-al) and 2-HOT +17:3-al, and after 21 days the diameters of moss colonies were measured. The results revealed a reduction in the colony diameter of 33% and 50%, when tissues were grown with 17:3-al or 2-HOT + 17:3-al, respectively, compared to control colonies (Figure 5A). No clear difference in colony diameter was observed when only 2-HOT was included in the medium (Figure 5A). Moss colonies grown in the presence of 17:3-al or 2-HOT + 17:3-al had less protonemal tissue with less extending protonemal filaments compared to control colonies (Figure 5B). To this end, we decided to generate Ppα-DOX overexpressing lines and knockout *Ppα-dox* mutants by homologous recombination to analyze in more detail the possible role played by Ppα-DOX-derived oxylipins in moss development. After transformation one stable overexpressing line, pUBI:Ppα-DOX-3, with 51% increase in α-DOX activity compared to wild-type plants was selected (Additional file 5). Since Southern blot analysis revealed that the knockout lines obtained had multiple insertions of the construct (data not shown), one knockout line (*Ppα-dox-2*) with null Ppα-DOX activity was selected. The Ppα-DOX-GUS-12 line was included for further studies since Ppα-DOX was disrupted in this line, having only one insertion, and no α-DOX activity (Additional files 3 and 5). Haploidy of all lines was confirmed by measurement of nuclear DNA content (data not shown). Both knockout lines, *Ppα-dox-2* and Ppα-DOX-GUS-12, were phenotypically indistinguishable from each other and behave similarly in all our experiments and therefore only the data of *Ppα-dox-2* are shown. No morphological abnormalities during the juvenile or adult gametophytic phases were detected in *Ppα-dox-2* (Figure 5D and Additional file 5), indicating that Ppα-DOX is not required for morphogenesis. The general architecture of the leafy shoot was unaffected in pUBI:Ppα-DOX-3 (Additional file 5), and no alteration in sporophyte formation or spore viability was observed in the different genotypes compared to wild-type plants (data not shown). However, moss colonies of the overexpressing pUBI:Ppα-DOX-3 line were clearly smaller, with a reduction in colony diameter of 40% compared to wild-type colonies (Figure 5C). Overexpressing pUBI:Ppα-DOX-3 colonies had less protonemal tissue with less extended protonemal filaments compared to wild-type colonies (Figure 5D), similar to wild-type colonies grown in the presence of 17:3-al or 17:3-al + 2-HOT (Figure 5B). The main Ppα-DOX product measured in wild-type and overexpressing pUBI:Ppα-DOX-3 tissues, when palmitic acid (16:0) is used as substrate, is the aldehyde pentadecanal (15:3-al) (Additional file 5). This result together with the reduced colony diameter observed in wild-type colonies grown in the presence of the aldehyde 17:3-al, suggest that Ppα-DOX-derived aldehydes are probably the oxylipins responsible for reduced growth.

### Effects of α-DOX-derived oxylipins on protonemal development

**Figure 6:**
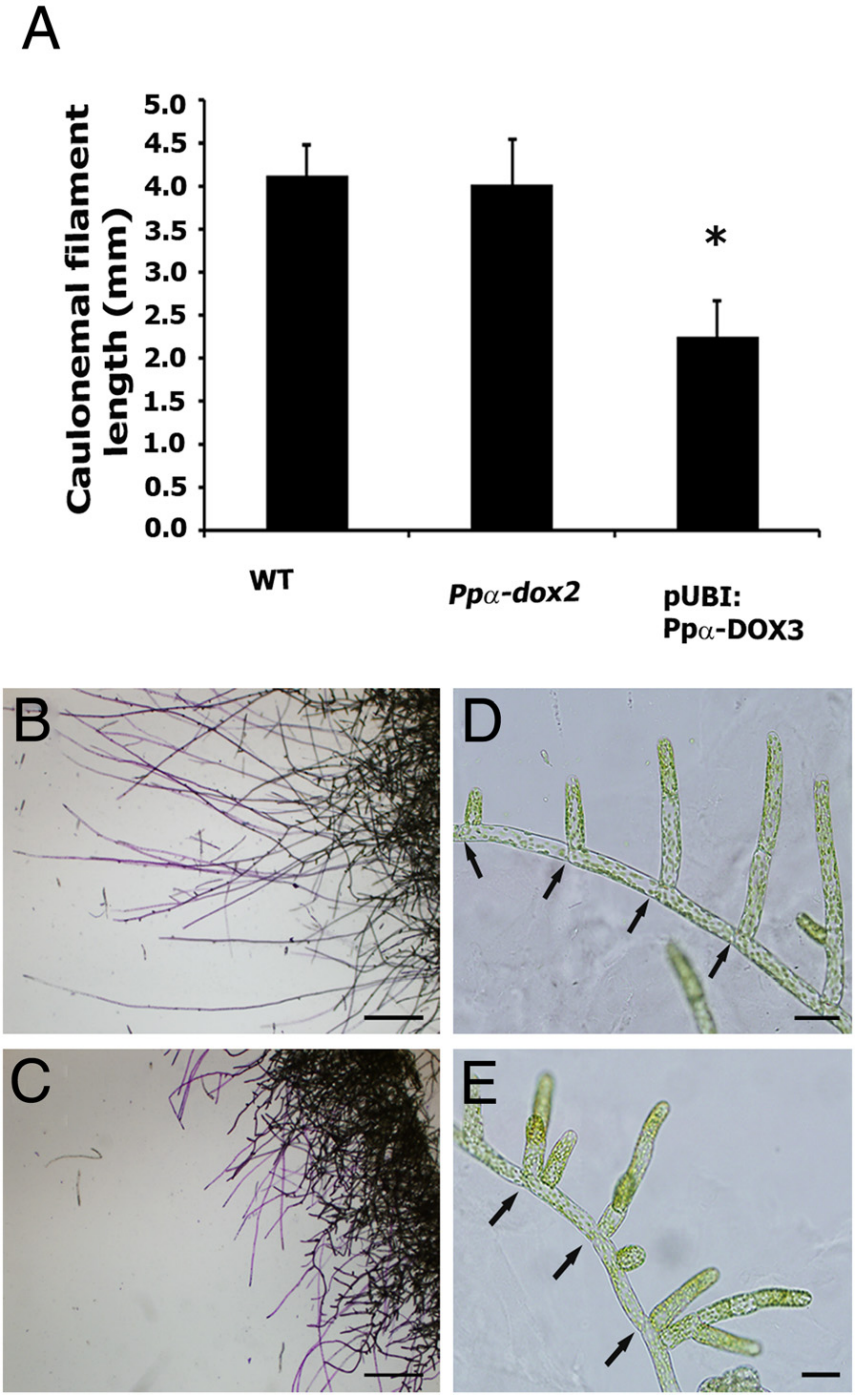
**Effect of α-DOX-derived oxylipins on caulonemal development. (A)** Values of average caulonemal filament length (in millimeters) measured in wild-type (WT), knockout line *Ppα-dox-2* and overexpressing line pUBI:PpαDOX-3 colonies grown for 10 days in caulonemal induction conditions. Results and standard deviation corresponding to 16 colonies per sample are shown. **(B)** Border of a WT colony and **(C)** Border of a pUBI:Ppα-DOX-3 colony grown in caulonemal induction conditions showing toluidine blue stained caulonemal filaments. **(D)** Wild-type protonemal filaments at the border of a colony grown in BCDAT medium. **(E)** pUBI:PpαDOX-3 protonemal filaments at the border of a colony grown in BCDAT medium. Asterisk for caulonemal filament length from pUBI:Ppα-DOX-3 colonies, indicate that the values are significantly different from caulonemal filament length from WT plants according to Kruskal–Wallis test: P <0.001. Arrows in **D** and **E** indicate septa between cells. Scale bars represent 0,1 cm in **B-C** and 20 μm in **D-E**.

Protonemal tissue initially consists of chloronemal cells with characteristic perpendicular cross walls and a high density of chloroplasts. From chloronemal filaments caulonemal cells arise subsequently with oblique cross walls and low density of chloroplasts. In turn, branching of caulonemal cells give rise to new chloronemal cells developing secondary chloronemal filaments [35]. To further analyze the effect of oxylipins on protonemal growth, we looked in more detail at caulonemal and chloronemal filament growth in the different lines and compared it with wild-type tissues. Since caulonemal filaments are responsible for radial growth [35], and could therefore affect moss colony size, we induced caulonemal formation and measured length of the filaments. The result showed that the overexpressing pUBI:Ppα-DOX-3 line has a significant reduction in the length of caulonemal filaments compared to wild-type plants (Figure 6A-C), which correlate with the reduced protonemal filament extension observed in Figure 5. The knockout line *Ppα-dox-2* did not reveal any difference in caulonemal filament length compared to wild-type colonies (Figure 6A). Wild-type secondary chloronemal filaments growing from caulonema showed a typical branching pattern under normal growth conditions (Figure 6D), while pUBI:Ppα-DOX-3 developed altered branching with two or more secondary chloronemal cells arising from one caulonemal cell (Figure 6E). Protonemal tissues grown in the presence of 17:3-al or 2-HOT + 17:3-al had only a few protruded caulonemal filaments, and most of the filaments were shorter compared to wild-type colonies (Figure 5B), similar to what was observed at the border of pUBI:Ppα-DOX-3 moss colonies (Figure 5D). The sizes of chloronemal cells in wild-type, *Ppα-dox-2* and pUBI:Ppα-DOX-3 tissues were also analyzed in chloronema inducing conditions (Additional file 6). When a distribution of chloronemal cell sizes in groups was performed, pUBI:Ppα-DOX-3 tissues had a higher proportion of cells with smaller sizes compared to wild-type and *Ppα-dox-2* cells (Additional file 6). Sizes of chloronemal cells were also evaluated in wild-type tissues grown under chloronema induction conditions in the presence of Ppα-DOX-derived oxylipins. We could observe a clear reduction in cell size of chloronemal cells, which corresponded to 41% and 48%, with 17:3-al and 2-HOT + 17:3-al respectively, compared to control cells (Additional file 6). Chloronemal cell sizes of tissues grown with 2-HOT were similar to control cells (Additional file 6). In addition, some alterations in chloronemal cell division were observed when tissues were grown with 17:3-al and 2-HOT + 17:3-al, as evidenced by the presence of cells with abnormal positioning of cross walls and filaments that did not have one typical apical cell (Additional file 6). Taken together, the results show that α-DOX-derived oxylipins, principally the aldehydes, alter moss development by reducing protonemal tissues formation, due to less protruded caulonemal filaments, reduced caulonemal filaments length and reduced chloronemal cell size, which leads to decreased colony diameter. In addition, incubation of wild-type tissues with 17:3-al or a combination of 17:3-al and 2-HOT leads to irregular chloronemal cell divisions.

### Effect of Ppα-DOX-derived oxylipins on cell death caused by *Pectobacterium carotovorum* elicitors

**Figure 7:**
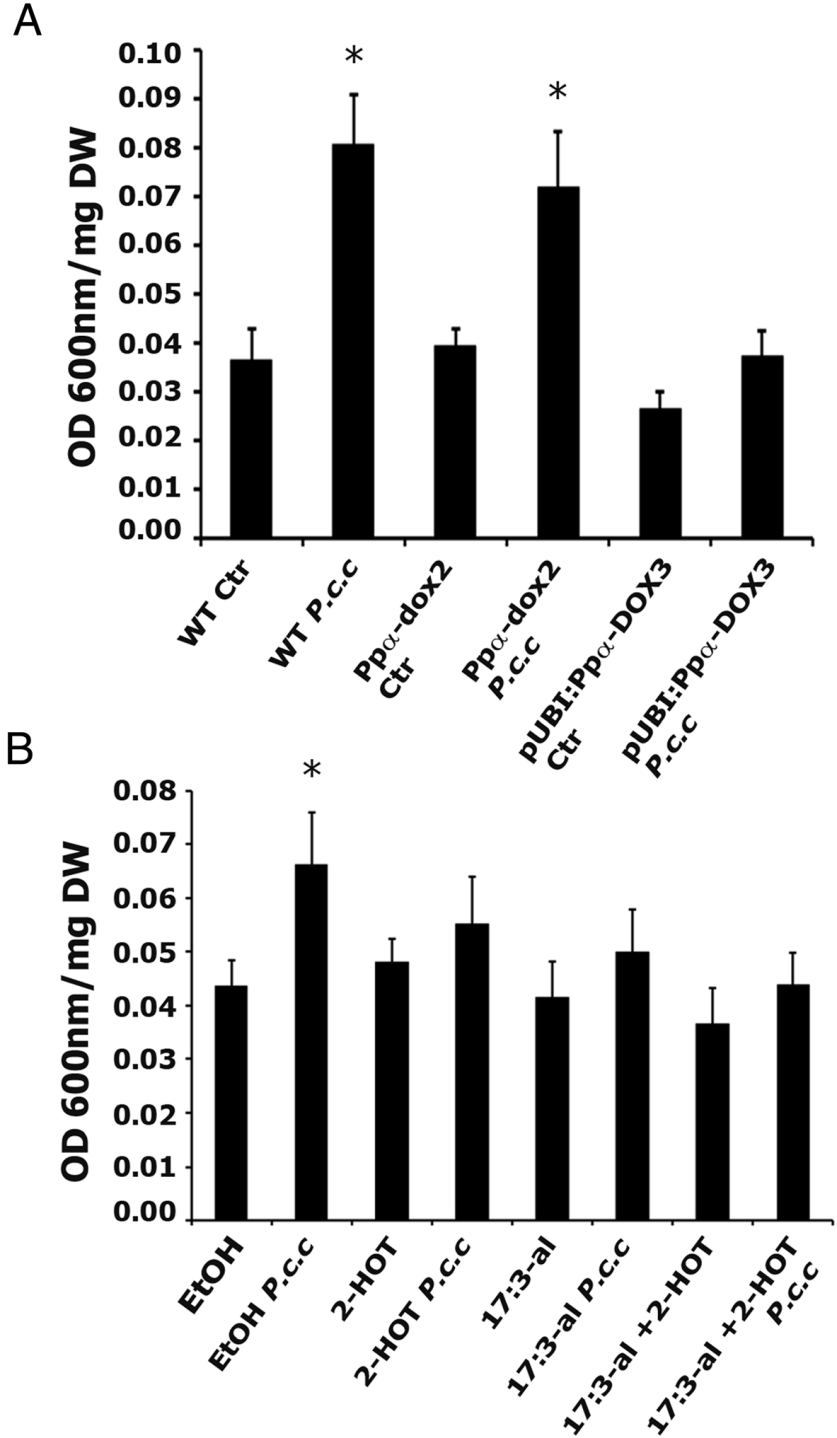
**Cell death in response to** ***P.c. carotovorum*** **elicitors in the different Ppα-DOX genotypes and in wild-type plants pretreated with oxylipins. (A)** Cell death measurements by Evans blue staining in wild-type (WT), *Ppα-dox-2* and pUBI:Ppα-DOX-3 colonies treated with elicitors of *P.c. carotovorum* (*P.c.c*) compared to water-treated colonies (Ctr). **(B)** Cell death in WT colonies treated for 2 days with 50 μM oxylipins was compared with the cell death generated in WT colonies treated for 1 day with 50 μM oxylipins and 1 additional day with 50 μM oxylipins + *P.c.c* elicitors by Evans blue staining. As control 0.5% ethanol was used. The values shown are means from one representative technical replicate. Error bars indicate SD (n = 8). Three biological replicates were carried out for each experiment with similar results. Significant differences of at least 0.05 confidence level between water-treated and elicitor-treated wild-type, *Ppα-dox-2* knockout line or pUBI:Ppα-DOX-3 plants in **(A)** or between oxylipins-treated and oxylipins + *P.c.c* elicitors-treated wild-type plants **(B)** are marked by *.

In order to analyze the possible role of Ppα-DOX in plant defense, cell death was measured by Evans blue staining in wild-type, the knockout line *Ppα-dox-2* and the overexpressing line pUBI:Ppα-DOX-3 after treating moss colonies for 1 day with elicitors of *P.c. carotovorum* and compared with water-treated colonies of the corresponding genotype. The results show that the knockout line *Ppα-dox-2* had similar cell death values after elicitor treatment compared to wild-type colonies, and both increased significantly compared to the corresponding water-treated colonies. In contrast, pUBI:Ppα-DOX-3 showed less cell death which did not increase significantly compared to water-treated pUBI:Ppα-DOX-3 colonies (Figure 7A). To further analyze the protective effect of Ppα-DOX derived oxylipins against damage caused by elicitors of *P.c. carotovorum*, the increase in cell death after treating moss colonies for 1 day with these elicitors was analyzed in control plants (pre-treated for 1 day with ethanol) and compared with plants pre-treated for 1 day with 50 μM 2-HOT, 50 μM 17:3-al or 50 μM 17:3-al + 50 μM 2-HOT (Figure 7B). The results show that while cell death caused by elicitors of *P.c. carotovorum* increased after pre-treating plant with ethanol, no significant increase in cell death could be observed when tissues were pre-treated with 2-HOT, 17:3-al, or a combination of 17:3-al and 2-HOT, suggesting that they protect tissues against cell damage caused by these elicitors.

## Discussion

### α-Dioxygenases are present in primitive land plants

Ppα-DOX catalyzed the oxygenation of fatty acids to synthesize the same products as α-DOX1 and α-DOX2 from flowering plants, including the 2-hydroxy fatty acid and the corresponding one-carbon atom chain shortened aldehyde [4,21]. Amino acid residues involved in heme binding (His-165 and His-389 in Atα-DOX1), and the catalytic tyrosine (Tyr-386 in Atα-DOX1) are conserved among flowering plant α-DOXs and Ppα-DOX [1,20,21,40]. Like Atα-DOX1, recombinant Ppα-DOX has a substrate preference for linolenic, linoleic and palmitic acid [40,41], while α-DOX2 from tomato and *A. thaliana* have a broader substrate specificity [21]. Phylogenetic analysis shows that Ppα-DOX belongs to the basal α-dioxygenase cluster along with its *Selaginella moellendorffii* [42] homologue, while flowering plant α-DOXs form a separate clade with two independent clusters, one containing α-DOX1 proteins and a second cluster containing α-DOX2 proteins [17,21]. Multicellular algae also have a putative α-DOX, evidenced by the presence of 2-hydroxypalmitic acid in *Ulva pertusa* [43]. Sequence data placed a putative α-DOX of the multicellular algae *Volvox carteri* in a unique cluster separated from plant α-dioxygenases. No putative α-DOX gene could be found in the unicellular green algae *Chlamydomonas reinhardtii*, suggesting that α-DOX proteins originated in multicellular algae. While more primitive land plants, including *P. patens* and *Selaginella moellendorffii*, have only one encoding α-DOX gene, most flowering plants have more than one α-DOX gene, which have probably specialized and acquired distinct biological functions during plant evolution.

### Ppα-DOX is highly expressed in mitotically active undifferentiated apical protonemal cells and in auxin producing differentiated gametophore cells

Ppα-DOX-GUS fusion proteins accumulate in tips of protonemal filaments and rhizoids, where the highest expression occurs in apical cells. These apical cells function as stem cells and divide continuously, producing a new apical stem cell and a differentiated subapical cell, allowing moss to grow by tip growth [44-46]. Ppα-DOX-GUS expression was also observed in other types of mitotically active cells, including dividing cells from detached leaves that readily reprogram, reenter the cell cycle and change their cell fate to become chloronemal apical stem cells [38], which also accumulate Ppα-DOX. Distal protonemal cells from regenerating protoplasts also expressed Ppα-DOX, with maximum GUS accumulation in apical cells. Thus, Ppα-DOX is highly expressed in mitotically active cells, suggesting that the oxylipins produced could play a role in undifferentiated protonemal apical cells and are less important in protonemal cells that have stopped dividing or have acquired cell fate.

Ppα-DOX expression is induced in leaves and cauloids of gametohpores after auxin application and Ppα-DOX-GUS accumulation at the basal and the apical part of the gametophore cauloid correlated with the two main locations of auxin occurrence which are sites of high levels of cell division [32,33]. Consistently, Ppα-DOX-GUS expression pattern in rhizoids and axillary hairs coincided with PpSHI2-GUS accumulation in gametophores of reporter lines which detect sites of auxin synthesis [32], and is similar to pGmGH3-GUS reporter lines which detect sites of auxin activity and response [33,34]. Expression of PpSHI2, which is a positive regulator of auxin biosynthesis, is detected only along the whole caulonema independent of the age, position or mitotic activity of the cells [32]. In addition, Aoyama et al. [47] have proposed the existence of a local loss of auxin in protonemal apical cells that might serve as a cue during cell fate determination. These findings suggest that additional signal(s) in protonemal cells, particularly in apical cells, contribute to Ppα-DOX expression.

### α-DOX-derived aldehydes induce alterations in differentiated cells of protonemal filaments

Ppα-DOX knockout mutants did not show any apparent developmental phenotype in the gametophyte compared to wild-type plants, suggesting that Ppα-DOX is not required for correct tip growth and morphogenesis in *P. patens*. Knocking-out α-DOX2 in flowering plants may or may not have an effect on development depending on the plant species [17,21]. While *A. thaliana* α-DOX2 mutant has no developmental alterations, tomato α-DOX2 mutant and *N. attenuata* co-suppressed α-DOX1 and α-DOX2 plants have a stunted phenotype [17,21]. Interestingly, moss colonies of the overexpressing pUBI:Ppα-DOX-3 line, with significantly higher Ppα-DOX activity compared to wild-type plants, and moss colonies of wild-type tissues grown in the presence of Ppα-DOX-derived oxylipins, principally 17:3-al, are smaller and have less protonemal tissues. This morphological effect is due to less protruded caulonemal filaments, reduced caulonemal filaments length and a higher proportion of chloronemal cells with reduced size. Since 17:3-al reduces protonemal growth, and aldehydes are the main Ppα-DOX-derived oxylipins accumulating in moss tissues, we consider that overexpressing lines with higher α-DOX activity than pUBI:Ppα-DOX-3 are difficult to select due to their small size compared to other transformants with less α-DOX activity. Thus, to ensure a proper development of wild-type moss tissues, Ppα-DOX production and/or activity are probably tightly regulated.

In *P. patens*, other oxylipins including jasmonates and OPDA also reduce colony size [26], although the mechanism underlying this phenomenon is unknown. In flowering plants jasmonates and OPDA arrest root growth and retard seedling growth [8,48,49]. These oxylipins can inhibit cell division by preventing cell cycle progression [50,51], and repressing mitosis-phase genes [52]. Jasmonate has a negative effect on *A. thaliana* leaf growth by reducing cell number and cell size, associated to reduced expression of the cell cycle regulator cyclin-dependent protein kinase CYCB1;1 [53]. Jasmonate also inhibits *A. thaliana* root length by reducing both cell number and cell length, involving differentiation of columella stem cell in the root meristem and irregular division of quiescent cells which are mitotically inactive [54]. In *P. patens* we observed an expression pattern for Ppα-DOX-GUS proteins with high expression in undifferentiated mitotically active protonemal apical cells, lower expression levels in differentiated subapical cells and no expression in the remaining older proximate differentiated protonemal cells. Constitutive production or higher than normal levels of Ppα-DOX-derived oxylipins, principally 17:3-al, in differentiated protonemal cells lead probably to reduced cell size and irregular divisions of these mitotically inactive cells. Ppα-DOX-derived oxylipins could also promote cell differentiation since Ppα-DOX-GUS proteins accumulate in reprogrammed cells of detached leaves which have reentered the cell cycle, divided and changed their cell fate to produce chloronema apical cells [38]. Thus, Ppα-DOX could contribute directly or indirectly to the fine-tuning of cell proliferation and differentiation in *P. patens*.

### α-DOX-derived oxylipins protect moss tissues against cellular damage caused by elicitors of *P.c. carotovorum*

α-DOX1 of flowering plants are induced after pathogen infection and herbivore attack and play a role in the defense response against this type of stress [11,13-15,17,18]. In this work, we show that transcript levels and Ppα-DOX activity increase after treatment with elicitors of *P.c. carotovorum* and spores of *B. cinerea*, both of which induce a hypersensitive response (HR) in flowering plants and *P. patens* [24,26,55,56]. Similarly, in *A. thaliana* and *N. tabacum*, transcriptional up-regulation of α-DOX1 is higher when plants are infected with *Pseudomonas* strains that induce an HR response [11,13]. Under normal conditions, Ppα-DOX accumulates in tips of protonemal filaments and rhizoids, which are the cells that are probably more exposed to soil-borne pathogen infection and might represent, together with other defense mechanisms, a permanent system of protection. Ppα-DOX is also expressed in axillary hairs, which in some moss species secrete a mucilage [57], that may protect young leaves from desiccation [58], and could also have a function in defense responses against pathogens. Ppα-DOX expression increases in protonemal tissues and leaves treated with elicitors of the harpin producing *P.c. carotovorum* strain or infected with *B. cinerea*. Interestingly, the Ppα-DOX-GUS accumulation pattern in cells surrounding *B. cinerea* infected cells in *P. patens* leaves was similar to *A. thaliana* α-DOX1 expression in leaves where GUS accumulated in cells surrounding tissues infected with a virulent *P. syringae* strain [13]. A possible role in protecting plant tissues against oxidative stress and cell death generated by pathogens is proposed for *A. thaliana* α-DOX1 [13,16], and Ppα-DOX could play similar functions in moss. Recently, García-Marcos et al. [59] have demonstrated that α-DOX1 from *N. benthamiana* positively regulates programmed cell death during infections with different viruses. While *A. thaliana* α-DOX1 is essential for an efficient defense response against pathogens [13], Ppα-DOX is not required and similar damage is caused by *B. cinerea* or elicitors of *P.c. carotovorum* in the knockout lines compared to wild-type plants. However, overexpression of Ppα-DOX and treatments with α-DOX-derived oxylipins lead to less cellular damage caused by elicitors of *P.c. carotovoum*, which contains cell wall degrading enzymes and the HR inducing harpin protein [55]. Consistently, *A. thaliana* plants overproducing α-DOX1 showed less cellular damage to *P. syringae* infection [13], and a reduction in the severity of the symptoms against this bacteria was evidenced when tobacco plants were pre-treated with 2-HOT [15], or when *A. thaliana* plants were pre-treated with 17:3-al or 2-HOT [16]. The mechanisms by which plant α-DOX-derived oxylipins protect plant tissues against pathogen infection are poorly understood. Recently, Shimada et al. [5] have demonstrated that after pathogen infection α-DOX1 and a caleosin (CLO3) from *A. thaliana* are recruited to oil bodies of areas surrounding the site of infection. Interestingly, Atα-DOX1 and AtCLO3 together favored the production of the stable oxylipin 2-HOT, which has anti-fungal activity against members of the genus *Colletotrichum* [5]. In addition, α-DOX-derived oxylipins could also play a role in the activation of genes encoding proteins involved in hormonal signaling [16], oxidative stress scavenging, cell death protection and/or inducing protein with antimicrobial activities.

## Conclusions

Here, we show that the unique α-DOX gene of the moss *P. patens* shares both functions of flowering plants α-DOX1 and α-DOX2 and participates in development and in the defense response. Thus, α-DOX from flowering plants could have originated by duplication and successive functional diversification after the divergence from bryophytes. Although Ppα-DOX is not crucial for development and defense against elicitors of *P.c. carotovorum*, higher than normal levels of oxylipins result in protonema developmental alterations, suggesting that oxylipin production is tightly regulated. Ppα-DOX expression patterns together with phenotypic analysis also suggest that the derived oxylipins could participate directly or indirectly in cell proliferation and differentiation, as well as in protecting moss tissues damaged by cell wall degrading enzymes and harpin of *P.c. carotovorum*. Here, we have modified for the first time the levels of α-DOX-derived oxylipins in the plant *P. patens*, providing new tools for the understanding of the role played by α-DOX in development and the adaptation of plants to their environment.

## Methods

### Sequence alignment and phylogenetic analysis of plant α-dioxygenases

Full-length amino acid sequences from confirmed and putative α-DOX were retrieved from databases. Sequences were aligned with ClustalW [60] in a MEGA version 5.05 software [61] for subsequent phylogenetic analysis. Construction of phylogenetic trees was done using the Neighbor joining algorithm [62]. The percentage of replicate trees is shown on the branches and it is calculated in the bootstrap test (1000 replicates) for the associated taxa being clustered together.

### Plant material, growth conditions and treatments

*Physcomitrella patens* Gransden 2004 wild-type isolate was grown and maintained on cellophane overlaid BCDAT medium [63], at 22°C under a photoperiod of 16 h light and with a photon flux of 60 μmol m^−2^ sec^−1^. All plants were grown under these conditions unless indicated. Protonemal cultures and moss colonies were grown as described previously [25], and three-week-old colonies were used for all the experiments, unless indicated. The distal halves of gametophore leaves of 3 weeks old plants were excised with a razor blade and cultivated on BCDAT medium for 4–5 days to analyse cell division and formation of chloronemal apical stem cells. To examine the effects of exogenous auxin, moss colonies grown for 3 weeks on BCDAT medium covered with cellophane were transferred to plates containing BCDAT medium supplemented with 5 μM 1-naphthalene acetic acid (NAA) for 2 days.

### Oxylipins

The oxylipins 8(Z),11(Z),14(Z)-heptadecatrienal (17:3-al) and 2(R)-hydroxy-9(Z),12(Z),15(Z)-octadecatrienoic acid (2-HOT) were obtained as previously described [64]. Oxylipin concentrated stocks were prepared in 95% ethanol and diluted with water to reach the concentration used in these studies. [16,16,16-^2^H_3_]2-Hydroxy-16:0 were prepared by chemical oxygenation of [16,16,16-^2^H_3_]16:0 (Sigma-Aldrich Sweden, Stockholm) [65], and the methyloxime of [15,15,15-^2^H_3_]pentadecanal was obtained by periodate oxidation of the deuterated 2-hydroxy-16:0 followed by treatment with 30 mM O-methyl hydroxylamine hydrochloride in methanol.

### Colony size measurements of plant tissues grown in media containing oxylipins

Small pieces of protonema of 1 mm were harvested from protonemal cultures of the different genotypes and placed on fresh plates containing BCDAT medium without cellophane. Alternatively, wild-type tissues were also placed on medium containing 50 μM of 17:3-al, 50 μM 2-HOT or a combination of both. As control, plants were grown on plates containing 0.5% ethanol. For each genotype, and for wild-type colonies grown with each oxylipin, two plates were set up containing 16 colonies each. Plants were observed after 21 days and the diameter of each colony was recorded. The colony diameter was measured using GIMP 2.6 software. All experiments were repeated at least three times. To compare the significance of the differences between the diameters of the colonies a nonparametric Kruskal–Wallis multiple comparison test was performed using STATISTICA7 software.

### Pathogen inoculation and culture filtrates treatments

*Pectobacterium carotovorum* subsp. *carotovorum* (*P.c. carotovorum*) strain SCC1 [66] was propagated on Luria-Bertani (LB) medium at 28°C. Cell-free culture filtrates were prepared by growing bacteria in LB broth overnight, removing bacterial cells by centrifugation (10 min at 4000 g) and filter sterilizing the supernatant (0.2 μm pore size). This filter-sterilized supernatant containing the elicitors was applied by spraying the moss colonies. *Botrytis cinerea* was cultivated on 24 g/L potato dextrose agar (PDA) (Difco) at 22°C. *B. cinerea* was inoculated by spraying a 2 × 10^5^ spores/mL suspension in water as described in Ponce de León et al. [26]. Water application was used as control.

### Assay of α-dioxygenase activity and product analysis

The substrate specificity of recombinant Ppα-DOX was analyzed by oxygen consumption using a Clark-type oxygen electrode (Hansatech Instruments). High Five insect cell pellets containing Ppα-DOX (approximately 100 μg total protein) were thawed in 50 mL 0.1 M Tris, pH 7.4, passed five times through a 100-μL Hamilton syringe, and rapidly brought to room temperature. Total protein content was determined by the method of Bradford using cell homogenates prepared in 0.1 M Tris, pH 7.4, with 0.1% Triton X-100. The broken cell preparations were added to the measuring cell containing 1.5 mL 0.1 M Tris, pH 7.4, 100 μM fatty acid substrate, and 100 μM *tert*-butyl-hydroperoxide. Oxygen consumption was recorded at room temperature, and the rate of enzyme activity calculated as nmol oxygen consumed during the first minute per mg protein. For product analysis, incubates of palmitic, linoleic and linolenic acids were treated consecutively with O-methyl hydroxylamine/pyridine (23°C for 15 h), diazomethane in diethyl ether-methanol (9:1, v/v) (30 sec), and trimethylchlorosilane/hexamethyldisilazane/pyridine (2:1:2, v/v/v) (23°C for 15 min). The derivatized products were analyzed by GC-MS using a Hewlett-Packard model 5970B mass selective detector connected to a Hewlett-Packard model 5890 gas chromatograph equipped with a phenylmethylsiloxane capillary column (12 m, film thickness 0.33 μm). Helium at a flow rate of 25 cm/s was used as the carrier gas. The column temperature was raised from 120°C to 300°C at a rate of 10°C/min.

Ppα-DOX activity in plant extracts was analyzed by adding 0.3 g of fresh protonemal tissue to 3 mL of 0.1 M ice cold potassium phosphate buffer pH 7.4 and homogenizing the tissues at 4°C with a polytron (Kinematica, Germany). The homogenate was filtered through a gauze, and 1 mL of the homogenate was incubated at 23°C with 200 μM palmitic acid for 30 min. O-Methyl hydroxylamine hydrochloride solution (3 mL of a 30 mM solution in methanol) was added and the mixture kept at room temperature for 1 h. A standard solution (0.5 mL, containing 4.025 nmol of [16,16,16-^2^H_3_]-2-hydroxy-16:0 and 10.900 nmol [15,15,15-^2^H_3_]pentadecanal methyloxime) was added to each sample, and the Ppα-DOX products (pentadecanal and 2-hydroxy-palmitic acid) were analyzed by GC-MS following methyl-esterification and trimethylsilylation as described above. The instrument was operated in the selected ion monitoring mode using the mass spectral ions *m/z* 343 and 346 (unlabeled and labeled derivatized 2-hydroxy-16:0, respectively) and *m/z* 224 and 227 (unlabeled and labeled derivatized pentadecanal, respectively) (Additional file 2). Ppα-DOX activity is expressed as the sum of both oxylipins.

### α-DOX-GUS fusion

For the Ppα-DOX-GUS fusion construct we used the vector pTN83, generated by Nishiyama T, and acquired from the Physcobase clone collection (http://moss.nibb.ac.jp). A DNA fragment corresponding to bases 2497 to 3506 (from the ATG) of the Ppα-DOX genomic sequence containing part of exon 7 up to the last exon but lacking the stop codon, was amplified using the primers 5′DOX (cggcaaccgcgggcagtagc) containing a *Sac*II restriction site, and 3′DOX (ggcttctctggtgtctgattcc). The amplified fragment was phosphorylated with T4 polynucleotide Kinase, digested with *Sac*II and cloned in frame with the GUS gene into the SacII and Klenow DNA polymerase blunted *Bam*HI sites of the vector pTN83. Another fragment of 1022 bp downstream of the stop codon, including the 3′UTR of Ppα-DOX and the adjacent genomic sequence was amplified using the primers 5′DOXUTR (tgtcgttgatctcaagcttgtagag) and 3′DOXUTR (caatttcaccagttctctcgaggattc), which contained restriction sites for *Hin*dIII and *Xho*I, respectively. This fragment was cloned into *Hin*dIII and *Xho*I sites downstream of the *npt*II selection cassette of the vector. The resulting plasmid was linearized with *Kpn*I and used to generate the Ppα-DOX-GUS lines which were selected with 40 μg mL^−1^ G418. PCR genotyping of stable transformants was performed with the combination of primers DOX3F (accggttacatcctttgctg) and GUS-R3 (tcttgtaacgcgctttcccaccaacgctga), and 3′DOXr (gcgggatggtatcaactgtg) and sensenptII (ctacccgtgatattgctgaagagc), respectively. PpDOX-GUS lines were further analyzed by Southern blot analysis. *In situ* localization of GUS activity was performed as described by Peleman et al. [67]. Tissues were stained at 37°C for 24 hours before destaining in an increasing serial dilution of ethanol, mounted in water, visualized in an Olympus BX61 microscope (Shinjuku-ku, Tokyo, Japan), and images were captured with the MICROSUITE software package (Olympus). Results obtained with two selected lines (Ppα-DOX-GUS-2 and Ppα-DOX-GUS-12) are shown indistinctly since both reporter lines were phenotypically indistinguishable from each other and had identical expression patterns under all conditions used in this study.

### Ppα-DOX overexpression

Ppα-DOX cDNA was amplified from clone pdp14290 (RIKEN BioResource Center of Japan), using primers DOX1 (forward primer “gacagtgaattcttgcaggttgag”) and DOX2 (reverse primer “cagtctgctcgaggtcttcagg”) which contained restriction sites for *Eco*RI and *Xho*I, respectively. The corresponding fragment of 2000 bp was cloned into *Eco*RI and *Xho*I sites of the pENT vector, and transferred via LR clonase (Applied Biosystem) to a pTHUbi destination vector (kindly provided by Pierre-Francois Perroud, Washington University in St. Louis, USA) which drives gene expression using the constitutive maize ubiquitin promoter [68]. The generated vector pUBI:Ppα-DOX was digested with *Swa*I before transformation and targeted to the 108 locus where homologous recombination yields no detrimental phenotypes [69]. Stable transformants were selected on 25 μg mL^−1^ hygromycin. Levels of Ppα-DOX transcript accumulation of the selected transformants were assayed by Northern blot analysis.

### Ppα-DOX gene disruption

For Ppα-DOX gene disruption, the vector pUBW302 containing the *npt*II gene driven by the constitutive 35S promoter and the 3′UTR of the ocs gene was used [70]. The disruption construct contained a 827 bp genomic fragment from the 5′ region of the Ppα-DOX gene cloned upstream from the 35S promoter, and a 755 bp genomic fragment corresponding to the 3′ region of the gene inserted downstream of the ocs terminator signal. The genomic Ppα-DOX sequence (corresponding to 117–3826 of the genomic sequence) was obtained by PCR using the primer forward “gtaacgttgggtcagttg” and reverse “tctctacaggcttgagatc” and cloned into pBlueScript KS vector. The 5′ sequence (corresponding to 117–827 of the genomic sequence) was obtained digesting pBlueScriptPpα-DOX with *Sma*I and *Hin*dIII, and cloned into the pUBW302 vector previously digested with *Xho*I and treated with Klenow DNA polymerase to generate blunt ends, and subsequently digested with *Hin*dIII. The 3′ sequence of the gene (corresponding to 2960–3715 of the genomic sequence) was obtained by PCR amplification of the genomic DNA sequence using gene specific primers containing sequences for restriction enzymes *Xba*I (forward primer “tccgcaggaggctctagaattgttc”) and *Not*I (reverse primer “ggatccaacgcggccgcctctgg”), and cloned into *Xba*I and *Not*I sites of the pUBW302 vector. The resulting plasmid was linearized with *Kpn*I and used to generate the *Ppα-dox* knockout lines which were selected with 40 μg mL^−1^ G418. PCR genotyping of stable transformants was performed with the combination of primers DOXF (forward primer “gtaacgttgggtcagttg”) and 35Santisense (“ctttctctgtgttcttgatgcagttag”), or DOXR (reverse primer “tctctacaggcttgagatc”) and SensenptII (“ctacccgtgatattgctgaagagc”), respectively.

### *P. patens* protoplast preparation and transformation

Protoplast preparation and polyethylene glycol-mediated transformation of protoplasts was performed as described previously [71]. For protoplast regeneration studies, protoplasts were plated on BCDAT medium supplemented with 10 mM CaCl_2_ and 0.44 M mannitol, and regeneration was followed daily. For transformation, protoplasts (1×10^6^ protoplasts/mL) were incubated with at least 15 ug of plasmid DNA and plated on BCDAT medium supplemented with 10 mM CaCl_2_ and 0.44 M mannitol. After 7 days, protoplasts were transferred to BCDAT medium supplemented with the appropriate antibiotic selection. To select for stable transformants, regenerating protoplasts were cycled on and off antibiotic plates for three 1-week intervals. Tissues of plants showing growth after three weeks of selection were harvested and analyzed for the incorporation of the transgene.

### Flow cytometry to measure DNA content

Two-week-old colonies were chopped with a razor blade in a Petri dish with 1 ml of nuclei extraction buffer (WPB) containing 0,2 M Tris–HCl pH 7.5, 4 mM MgCl_2_, 2 mM EDTA Na_2_, 86 mM NaCl, 1% Triton X-100, 10 mM K_2_O_5_S_2_, and 1% PVP-10, and incubated for 15 min on ice. The resulting suspension was filtered through a 50 μm nylon mesh and incubated with 50 μl of Propidium Iodide (PI) (1 mg/mL, final concentration 50 μg/ml) and 50 μl of RNase A (1 mg/mL, final concentration 50 μg/ml) for 10 min at room temperature, to stain the DNA and to avoid double stranded RNA staining. A FACSVantage flow cytometer (Becton Dickinson, California, USA) equipped with an Innova 300 laser (Coherent, USA) tuned to emit light at 488 nm was used. Laser power was set to 100 mW and PI fluorescence was collected in FL2 using a 575/26 band pass filter. Chicken red blood cells (CRBC) and DNA QC particles (BD) were used to calibrate the flow cytometer and to optimize fluorescence detection as well as to check instrument linearity. Analysis of flow cytometer parameters were carried out with CELLQuest software (BD). Wild-type samples of *Physcomitrella patens* were used as an external control of DNA ploidy level.

### Southern blot analysis

Genomic DNA was extracted according to Dellaporta et al. [72] with an additional RNase treatment and phenol extraction using 3 plates of 14 d grown protonemal tissues. 10 μg of genomic DNA was digested with *Sty*I, separated in 1% agarose gels and transferred to nylon filters (Hybond-N+, Amersham, GE Healthcare) according to Sambrook et al. [73]. Membranes were prehybridized at 65°C in 6 × SCC, 0.5% SDS, 0.125 mg milk powder and 20 μg mL^−1^ denatured salmon sperm DNA. Hybridizations were performed at 65°C overnight. A *Sty*I restriction fragment containing part of the *npt*II gene from the selection cassette and part of the Ppα-DOX 3′UTR, was labeled with [α32 ^P^]-dCTP using the Rediprime II random priming labeling system (Amersham Pharmacia Biotech) and used as a probe. After hybridization, membranes were washed twice for 30 min at 65°C with 5 × SCC, 0.1% SDS and twice 30 min with 2 × SCC, 0.1% SDS. Subsequently, membranes were exposed on autoradiography film.

### Northern blot analysis

Total RNA was isolated from *P. patens* plants using standard procedures based on phenol/chloroform extraction followed by LiCl precipitation. Each sample consisted of 48 colonies. 10 μg of total RNA samples were separated, transferred to nylon membranes (Hybond-N+, Amersham, GE Healthcare) according to Sambrook et al. [73], and immobilized at 120°C for 30 min. *P. patens* α-DOX cDNA clone from RIKEN [74] was linearized with *Xho*I and used for digoxigenin-labeled RNA (DIG-RNA) probe synthesis using a DIG RNA labeling kit (Roche). Membranes were prehybridized at 50°C in a hybridization mix containing 50% formamide, 5 X SCC, 0.1% SDS, 1 mg/mL powder milk, and 20 μg mL^−1^ denatured salmon sperm, and hybridized at 50°C overnight with 100 ng per mL of a DIG-α-DOX riboprobe. Membranes were washed twice at room temperature for 15 min in 2 X SCC and 0.1% SDS, then washed twice at 62°C for 15 min in 0.5 X SCC and 0.1% SDS, and used directly for chemiluminiscent detection according to the manufacturer’s instructions. Subsequently, membranes were exposed on autoradiography film. Equal amounts of total RNA loadings was verified by addition of ethidium bromide to the samples and photographing under UV light after electrophoresis. Blots shown are representative examples of the results obtained in three independent experiments.

### Semi-quantitative RT-PCR analysis

For cDNA synthesis, 2 μg of total RNA was treated with DNase I (Thermo Scientific) according to manufacturer’s instructions. cDNA was synthesized from total RNA using RevertAid Reverse transcriptase (Thermo Scientific) and oligo (dT) according to the manufacturer’s protocol. From the resulting 25 μL of cDNA, 2 μL were used as a template for PCR analysis using gene specific primers. Ppα-DOX and Elongation Factor-1 alpha (PpEF) expression was analyzed with the combination of primers DOXF and DOXR, and EFF (forward primer “gaagcggcggagatgaac”) and EFR (reverse primer “acgtctgcctcgctctagc”), respectively. For the analysis of the fused Ppα-DOX-GUS transcript, the combination of primers DOXF and GUS-R3 was used. The PCR conditions were as follows: 33 (Ppα-DOX and Ppα-DOX-GUS ) or 26 (PpEF) cycles at 94°C for 30 s, 52°C (Ppα-DOX and Ppα-DOX-GUS) or 55°C (PpEF) for 40 s, and 72°C for 90 s. RT-PCR of PpEF was used as a control to monitor cDNA template amounts.

### Phenotypic analysis

Colony size was measured as described previously. For caulonemal filament induction assays, moss colonies were grown for 10 days in BCDAT medium, then transferred to BCDAT medium supplemented with 5 g/L glucose and grown in the dark for 10 additional days. Because of negative gravitropism, Petri dishes were kept upside-down for an easy observation of caulonemal filaments. For proper visualization of caulonemal filaments, moss colonies were stained for 1 min in 0.05% toluidine blue in citrate-citric acid buffer and rinsed in water to remove excess dye.

For chloronemal filament induction assays, protonemal tissue was subcultured at 7-day intervals on BCDAT medium, then protonemal tissues were diluted 1/20 in water, grown on BCD medium without ammonium tartrate and analyzed after 6 days of growth. Representative pictures are shown. Caulonemal filament length and chloronemal cell sizes were measured using GIMP 2.6 software. For chloronemal cell sizes measurements, apical cells were not included. Tissues were visualized in an Olympus IX81microscope, and images were captured with the CellF software package (Olympus).

### Cell death measurements

Cell death was measured by incubating moss colonies with 0.05% Evans blue and after 2 hours tissues were washed 4 times with deionized water to remove excess and unbound dye. Dye bound to dead cells was solubilized in 50% methanol with 1% SDS for 45 min at 50°C and the absorbance measured at 600 nm [75]. Each sample consisted of 4 colonies incubated in 6 mL of the mixture methanol/SDS. Eight samples were analyzed per experiment. Each experiment was repeated at least three times and expressed as OD/mg dry weight. Dry weight was measured after drying plant colonies for 18 hours at 65°C.

## Additional files

Additional file 1:
**α-Dioxygenase-catalyzed metabolism of palmitic acid in** ***P. patens*** **tissues.**
Additional file 2:
**Selected ion chromatograms in quantitative determination of pentadecanal and 2-hydroxy-16:0.** *Upper panel*, *m/z* 224 (unlabeled pentadecanal-methyloxime) and *m/z* 227 (internal standard of ^2^H_3_-pentadecanal-methyloxime), *lower panel*, *m/z* 343 (unlabeled 2-hydroxy-16:0 methyl ester/trimethylsilyl derivative) and *m/z* 346 (internal standard of ^2^H_3_-2-hydroxy-16:0 methyl ester/trimethylsilyl derivative). Additional file 3:
**Generation of Ppα-DOX-GUS lines.** **(A)** Genomic structure of the Ppα-DOX locus (top), Ppα-DOX-GUS construct (middle), and expected outcome of DNA integration in the Ppα-DOX locus (bottom). Boxes indicate exons and the lines between the boxes, introns. **(B)** PCR-based genotyping of the Ppα-DOX-GUS transformants with primers DOX3F+GUS-R3 for 5′ region insertion events and sensenptII+3′DOXr for 3′ region insertion events. Expected PCR fragments of 1968 bp and 1202 bp for the right and left borders respectively, are indicated with an arrow. A negative control PCR reaction without DNA was included for each set of primers (−). **(C)** Southern blot analysis for Ppα-DOX-GUS lines. Genomic DNA of the wild-type (WT) and of the Ppα-DOX-GUS-lines 2, 8, 11, 12, 13 and 14 were digested with StyI. Recognition sites for StyI and the probe used are indicated with an S and with a thick red line, respectively in **(A)**. The expected hybridization bands are indicated with a black arrow for wild-type DNA or with a red arrow for DNA of Ppα-DOX-GUS lines. **(D)** Ppα-DOX activity in wild-type (WT), Ppα-DOX-GUS-2 and Ppα-DOX-GUS-12 tissues treated with elicitors of *P.c. carotovorum* for 1 day. **(E)** PCR amplification of a wild-type copy of Ppα-DOX in the WT, Ppα-DOX-GUS-2 and Ppα-DOX-GUS-12. **(F)** GUS staining of gametophores and protonemal tissues of Ppα-DOX-GUS-2 and Ppα-DOX-GUS-12 lines. **(G)** Semi-quantitative RT-PCR showing levels of Ppα-DOX-GUS fused transcripts in lines Ppα-DOX-GUS-2 and Ppα-DOX-GUS-12. RNAs were harvested from 3-weeks-old colonies treated with water (C) or elicitors of *P.c. carotovorum* (*P.c.c*) for 1 day. The expected fragment of 2424 bp was amplified with primers DOXF and GUS-R3 in Ppα-DOX-GUS-12. The Elongation Factor-1 alpha (PpEF1) transcript was used as control for cDNA template amounts. uidA, *uidA* coding region; Ter, nopaline synthase polyadenylation signal; p35S, 35S promoter; nptII, nptII selection cassette. Additional file 4:
**Ppα-DOX-GUS accumulation in response to auxin treatment.** Border of a representative untreated Ppα-DOX-GUS colony **(A)**, and a Ppα-DOX-GUS colony treated for 2 days with 5 μM NAA **(B)**. Untreated Ppα-DOX-GUS gametophore **(C)**, and Ppα-DOX-GUS gametophore treated for 2 days with 5 μM NAA **(D)**. Scale bars: 0,1 cm in A-B and 300 μm in C-D. Additional file 5:
**Generation of overexpressing and knockout Ppα-DOX lines.** **(A)** Schematic representation of Ppα-DOX overexpressing construct using plasmid pTHUbi. **(B)** Genomic structure of Ppα-DOX locus (top), *Ppα-dox* disruption construct (middle), and expected outcome of construct integration leading to the generation of *Ppα-dox* knockout lines (bottom). Boxes indicate exons and the lines between the boxes, introns. **(C)** Transcript levels of Ppα-DOX in wild-type (WT) and pUBI:Ppα-DOX overexpressing lines. **(D)** PCR-based genotyping of the Ppα-DOX knockout lines with primers DOXF+35Santisense for 5′ region insertion events and sensenptII+DOXR for 3′region insertion events. The knockout lines *Ppα-dox-2* and *Ppα-dox-11* had the expected PCR product of 1080 bp only for the left border, while the fragment of 1150 corresponding to the right border could not be amplified. A negative control PCR reaction without DNA was included (−). **(E)** Semi-quantitative RT-PCR analysis of Ppα-DOX transcripts in WT and *Ppα-dox* knockout plants. The expected PCR product of 2062 bp was amplified with DOXF and DOXR primers in wild-type plants treated with water (C) and elicitors of *P.c. carotovorum* (*P.c.c*), and not in elicitor-treated *Ppα-dox* knockout lines, confirming the loss of Ppα-DOX transcripts. The Elongation Factor-1 alpha (PpEF1) transcript was used as control for cDNA template amounts. **(F)** Ppα-DOX activity in tissues of untreated WT and pUBI:Ppα-DOX overexpressing plants, and WT and *Ppα-dox* knockout lines treated with elicitors of *P.c. carotovorum* for 1 day. **(G)** Individual values of pentadecanal and 2-Hydroxy-16:0 leading to the total Ppα-DOX activity presented in E of WT, knockout line *Ppα-dox-2* and the overexpressing line pUBI:Ppα-DOX-3 tissues. **(H)** Phenotype of WT, *Ppα-dox-2* and pUBI:Ppα-DOX-3 gametophores stained with 0.05% toluidine blue. Scale bars represent 300 μm. pUBI, maize ubiquitin promoter; 108, 108 locus; attR1 and attR2, recombination sites; Ter, nopaline synthase polyadenylation signal; p35S, 35S promoter; Hyg, hygromycin resistance cassette; nptII, nptII selection cassette. Additional file 6:
**Effect of α-DOX-derived oxylipins on chloronemal development.** **(A)** Repartition of chloronemal cell length (in micrometers) in protonemal tissues grown on chloronemal induction conditions for 6 days. Black columns, pUBI:Ppα-DOX-3; dark gray columns, wild-type; light gray columns, *Ppα-dox-2*. **(B)** Values of average chloronemal cells length (in micrometers) measured in wild-type tissues grown for 6 days in 50 μM 17:3-al, 50 μM 2-HOT and 50 μM 2-HOT+ 50 μM 17:3-al-containing medium in comparison with control plants grown on 0.5% ethanol. Wild-type protonemal filaments showing typical phenotype after 6 days of growth on chloronemal induction conditions on 50 μM 17:3-al-containing BCD medium **(D)**, 50 μM 2-HOT+ 50 μM 17:3-al-containing BCD medium **(E)**, in comparison with control plants grown on 0.5% ethanol containing BCD medium **(C)**. Black arrows in C-E indicate septa between cells. Red arrows in D indicate abnormal cell division. Asterisks for chloronemal cells length grown for 6 days in 17:3-al or HOT+ 17:3-al-containing medium **(B)**, indicate that the values are significantly different from chloronemal cells length grown for 6 days in 0.5% ethanol, according to Kruskal–Wallis test: P <0.001. Scale bars: 20 μm.
