# Supplementary material for: The Physcomitrella patens unique alpha-dioxygenase participates in both developmental processes and defense responses
Source: BMC Plant Biol. 2015 Feb 12;15:45. doi: 10.1186/s12870-015-0439-z (PMC4334559; doi:10.1186/s12870-015-0439-z)
Supplement: Additional file 1: — α-Dioxygenase-catalyzed metabolism of palmitic acid in P. patens tissues. [file 12870_2015_439_MOESM1_ESM.pdf]

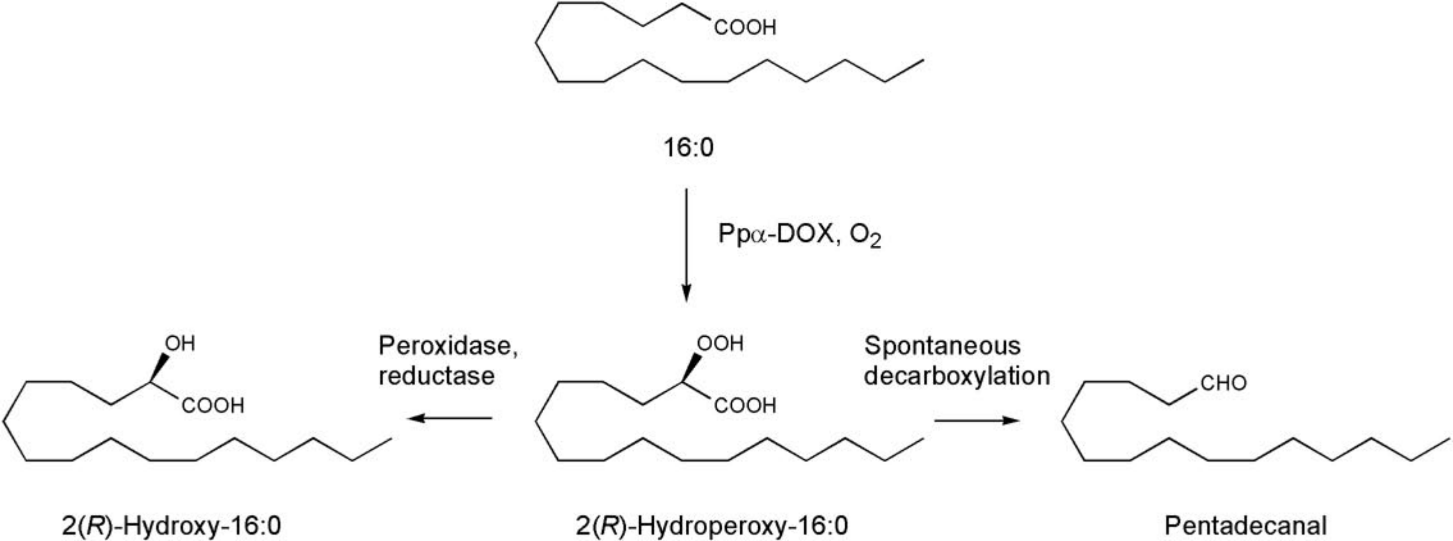
COOH
16:0
Ppα-DOX, O2
OH
COOH
Peroxidase,
reductase
OOH
COOH
Spontaneous
decarboxylation
CHO
2(R)-Hydroxy-16:0
2(R)-Hydroperoxy-16:0
Pentadecanal
