# Supplementary figures and images for: The Physcomitrella patens unique alpha-dioxygenase participates in both developmental processes and defense responses
Source: BMC Plant Biol. 2015 Feb 12;15:45. doi: 10.1186/s12870-015-0439-z (PMC4334559; doi:10.1186/s12870-015-0439-z)

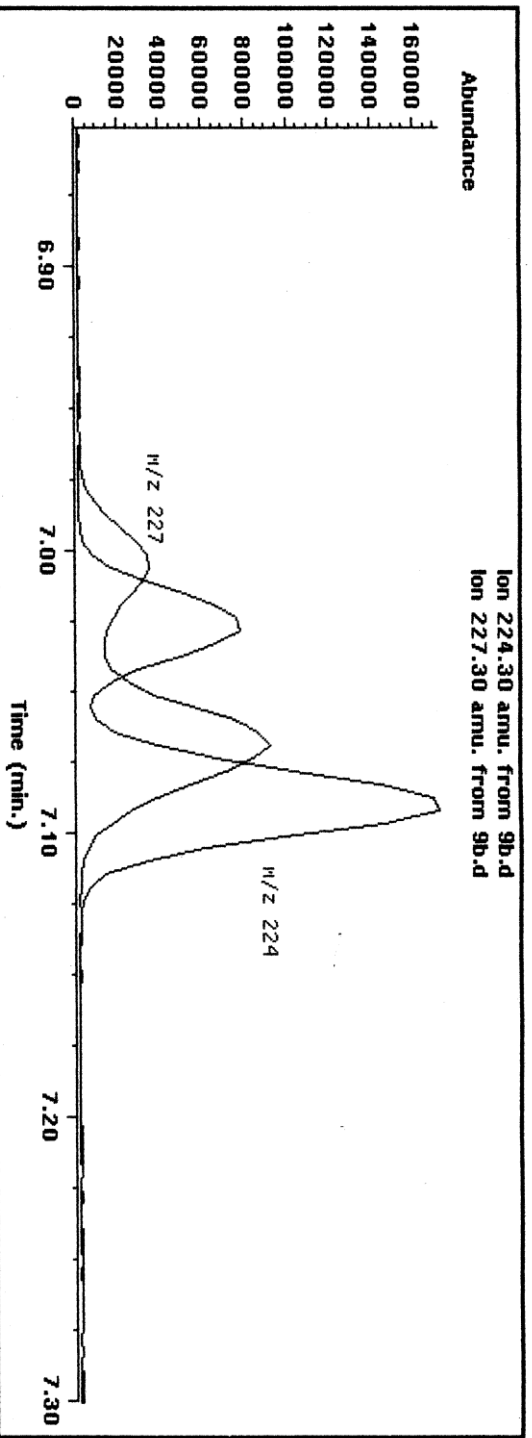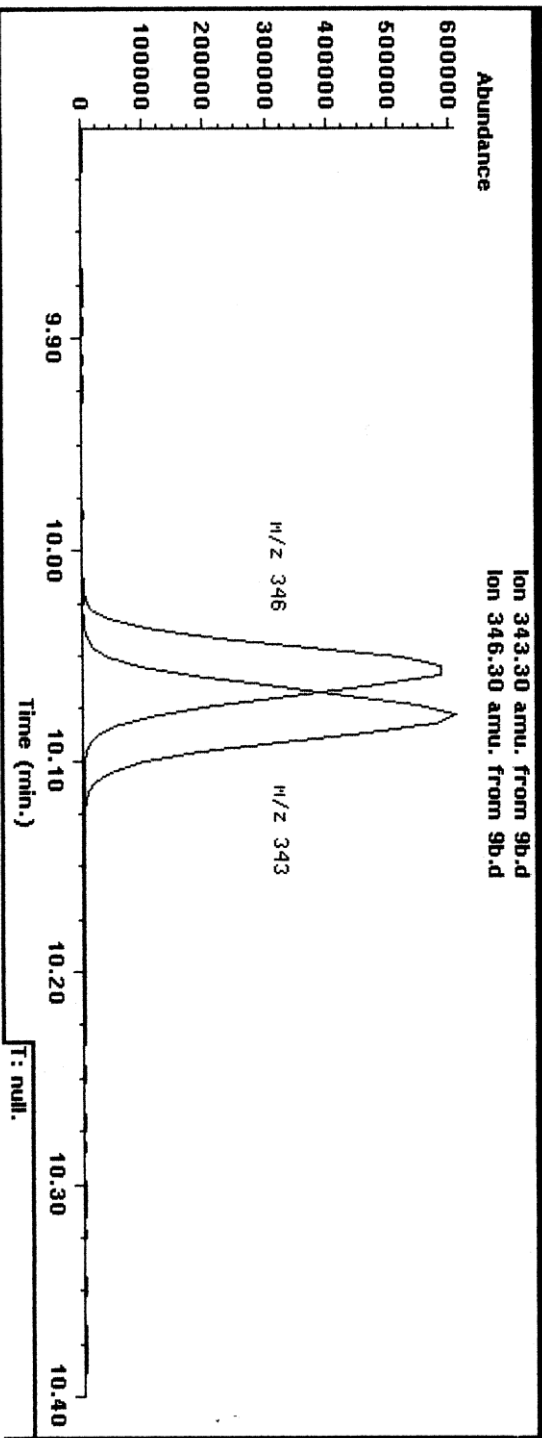

Supplement: Additional file 2: — Selected ion chromatograms in quantitative determination of pentadecanal and 2-hydroxy-16:0. Upper panel, m/z 224 (unlabeled pentadecanal-methyloxime) and m/z 227 (internal standard of 2H3-pentadecanal-methyloxime), lower panel, m/z 343 (unlabeled 2-hydroxy-16:0 methyl ester/trimethylsilyl derivative) and m/z 346 (internal standard of 2H3-2-hydroxy-16:0 methyl ester/trimethylsilyl derivative). [file 12870_2015_439_MOESM2_ESM.pdf]

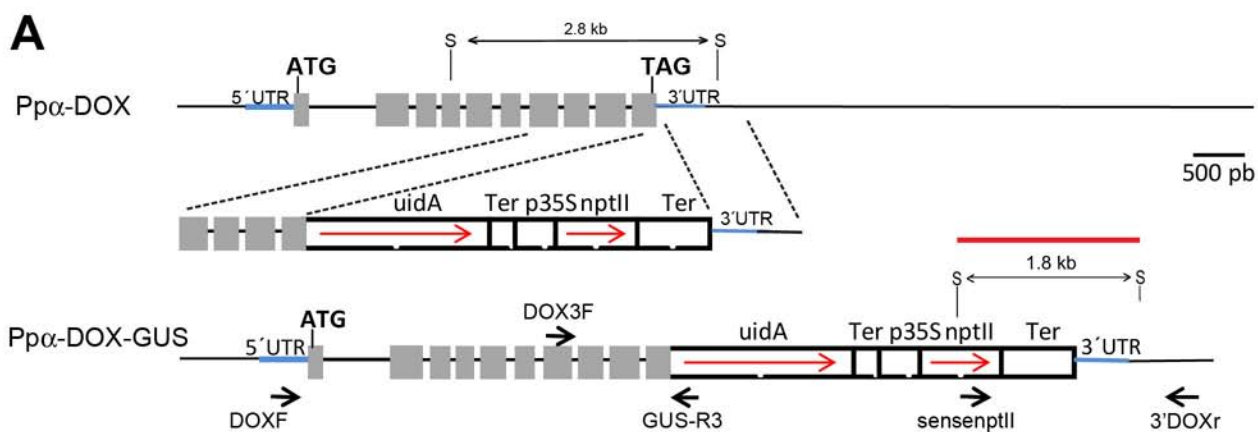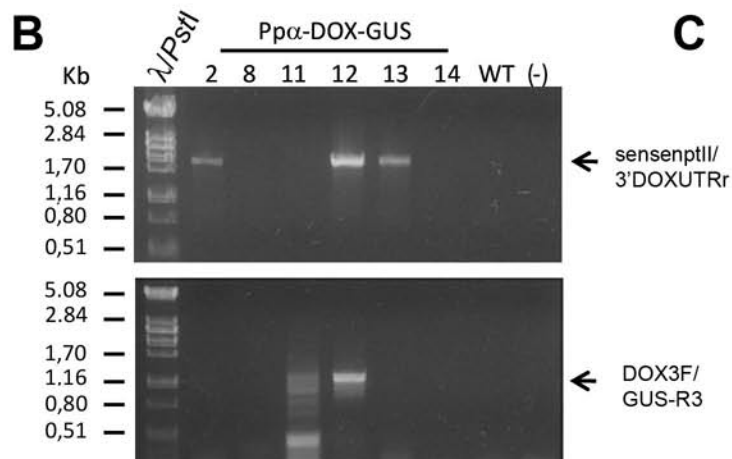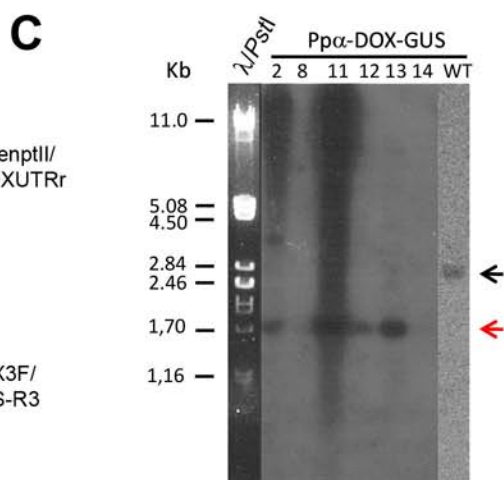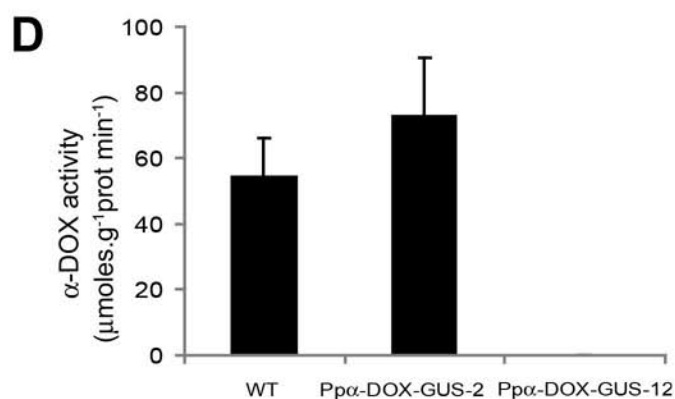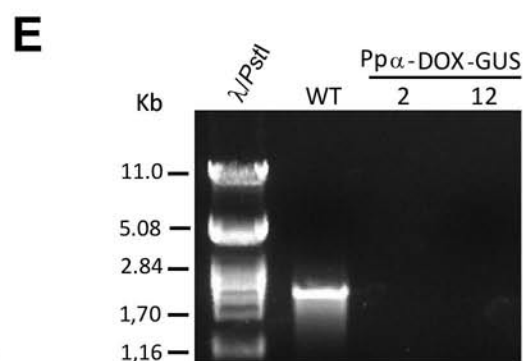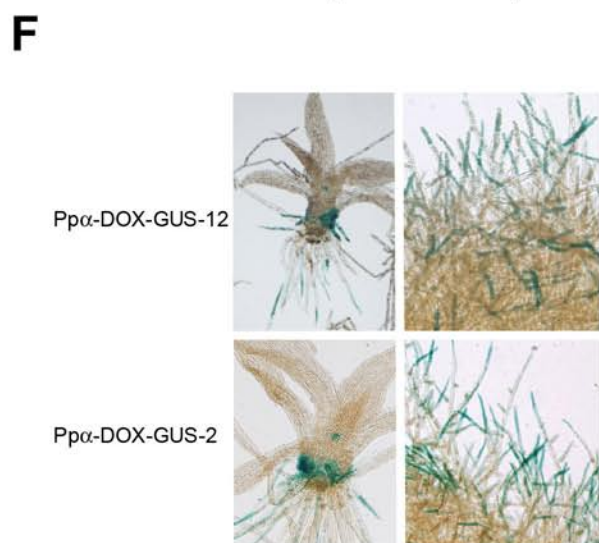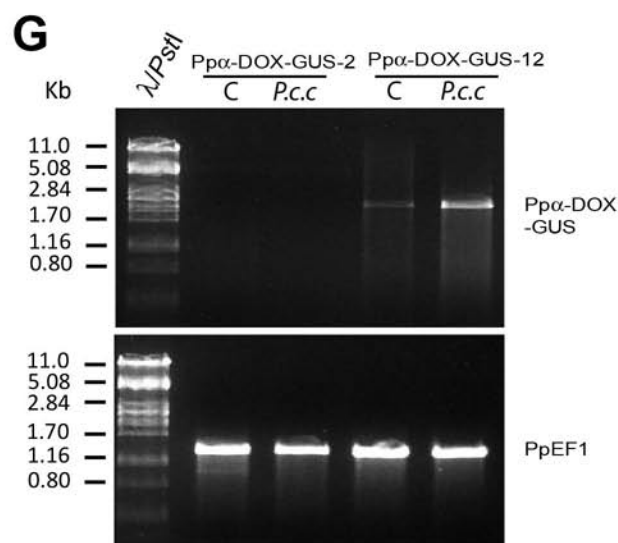

Supplement: Additional file 3: — Generation of Ppα-DOX-GUS lines. (A) Genomic structure of the Ppα-DOX locus (top), Ppα-DOX-GUS construct (middle), and expected outcome of DNA integration in the Ppα-DOX locus (bottom). Boxes indicate exons and the lines between the boxes, introns. (B) PCR-based genotyping of the Ppα-DOX-GUS transformants with primers DOX3F+GUS-R3 for 5′ region insertion events and sensenptII+3′DOXr for 3′ region insertion events. Expected PCR fragments of 1968 bp and 1202 bp for the right and left borders respectively, are indicated with an arrow. A negative control PCR reaction without DNA was included for each set of primers (−). (C) Southern blot analysis for Ppα-DOX-GUS lines. Genomic DNA of the wild-type (WT) and of the Ppα-DOX-GUS-lines 2, 8, 11, 12, 13 and 14 were digested with StyI. Recognition sites for StyI and the probe used are indicated with an S and with a thick red line, respectively in (A). The expected hybridization bands are indicated with a black arrow for wild-type DNA or with a red arrow for DNA of Ppα-DOX-GUS lines. (D) Ppα-DOX activity in wild-type (WT), Ppα-DOX-GUS-2 and Ppα-DOX-GUS-12 tissues treated with elicitors of P.c. carotovorum for 1 day. (E) PCR amplification of a wild-type copy of Ppα-DOX in the WT, Ppα-DOX-GUS-2 and Ppα-DOX-GUS-12. (F) GUS staining of gametophores and protonemal tissues of Ppα-DOX-GUS-2 and Ppα-DOX-GUS-12 lines. (G) Semi-quantitative RT-PCR showing levels of Ppα-DOX-GUS fused transcripts in lines Ppα-DOX-GUS-2 and Ppα-DOX-GUS-12. RNAs were harvested from 3-weeks-old colonies treated with water (C) or elicitors of P.c. carotovorum (P.c.c) for 1 day. The expected fragment of 2424 bp was amplified with primers DOXF and GUS-R3 in Ppα-DOX-GUS-12. The Elongation Factor-1 alpha (PpEF1) transcript was used as control for cDNA template amounts. uidA, uidA coding region; Ter, nopaline synthase polyadenylation signal; p35S, 35S promoter; nptII, nptII selection cassette. [file 12870_2015_439_MOESM3_ESM.pdf]

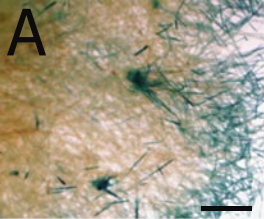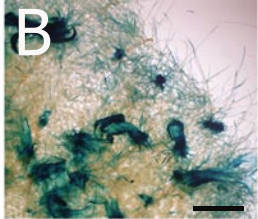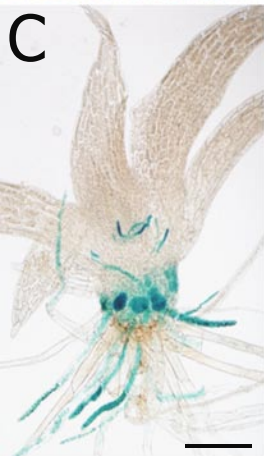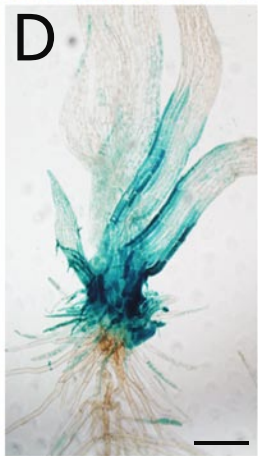

Supplement: Additional file 4: — Ppα-DOX-GUS accumulation in response to auxin treatment. Border of a representative untreated Ppα-DOX-GUS colony (A), and a Ppα-DOX-GUS colony treated for 2 days with 5 μM NAA (B). Untreated Ppα-DOX-GUS gametophore (C), and Ppα-DOX-GUS gametophore treated for 2 days with 5 μM NAA (D). Scale bars: 0,1 cm in A-B and 300 μm in C-D. [file 12870_2015_439_MOESM4_ESM.pdf]

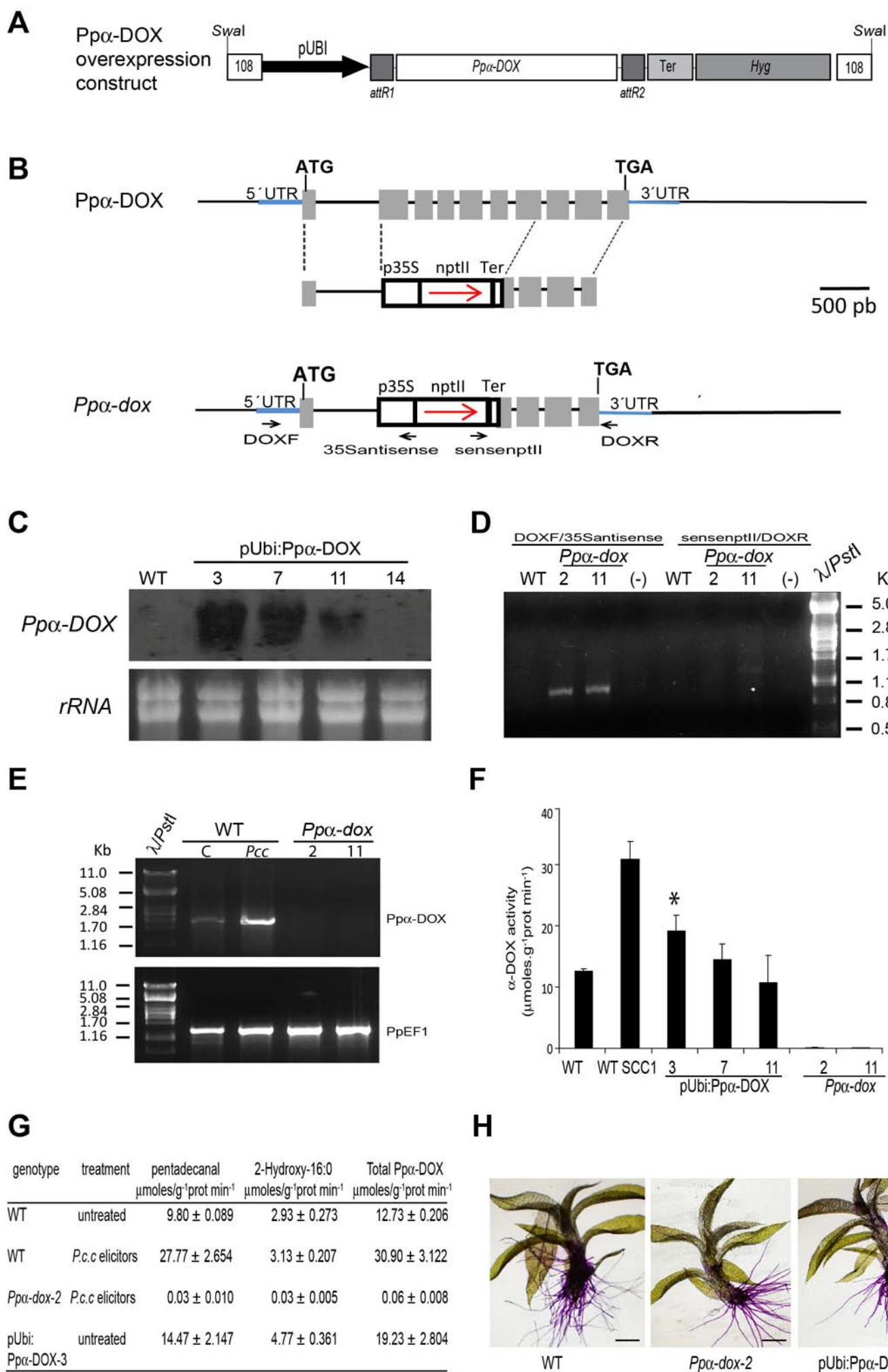

Supplement: Additional file 5: — Generation of overexpressing and knockout Ppα-DOX lines. (A) Schematic representation of Ppα-DOX overexpressing construct using plasmid pTHUbi. (B) Genomic structure of Ppα-DOX locus (top), Ppα-dox disruption construct (middle), and expected outcome of construct integration leading to the generation of Ppα-dox knockout lines (bottom). Boxes indicate exons and the lines between the boxes, introns. (C) Transcript levels of Ppα-DOX in wild-type (WT) and pUBI:Ppα-DOX overexpressing lines. (D) PCR-based genotyping of the Ppα-DOX knockout lines with primers DOXF+35Santisense for 5′ region insertion events and sensenptII+DOXR for 3′region insertion events. The knockout lines Ppα-dox-2 and Ppα-dox-11 had the expected PCR product of 1080 bp only for the left border, while the fragment of 1150 corresponding to the right border could not be amplified. A negative control PCR reaction without DNA was included (−). (E) Semi-quantitative RT-PCR analysis of Ppα-DOX transcripts in WT and Ppα-dox knockout plants. The expected PCR product of 2062 bp was amplified with DOXF and DOXR primers in wild-type plants treated with water (C) and elicitors of P.c. carotovorum (P.c.c), and not in elicitor-treated Ppα-dox knockout lines, confirming the loss of Ppα-DOX transcripts. The Elongation Factor-1 alpha (PpEF1) transcript was used as control for cDNA template amounts. (F) Ppα-DOX activity in tissues of untreated WT and pUBI:Ppα-DOX overexpressing plants, and WT and Ppα-dox knockout lines treated with elicitors of P.c. carotovorum for 1 day. (G) Individual values of pentadecanal and 2-Hydroxy-16:0 leading to the total Ppα-DOX activity presented in E of WT, knockout line Ppα-dox-2 and the overexpressing line pUBI:Ppα-DOX-3 tissues. (H) Phenotype of WT, Ppα-dox-2 and pUBI:Ppα-DOX-3 gametophores stained with 0.05% toluidine blue. Scale bars represent 300 μm. pUBI, maize ubiquitin promoter; 108, 108 locus; attR1 and attR2, recombination sites; Ter, nopaline synthase polyadenylation signal; p35S, [file 12870_2015_439_MOESM5_ESM.pdf]

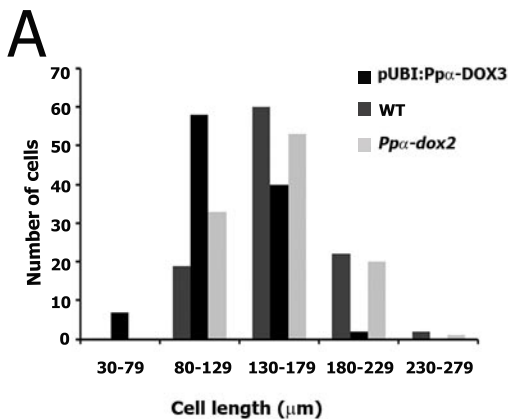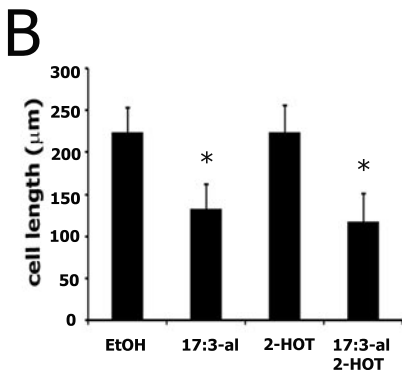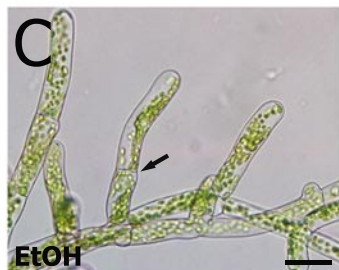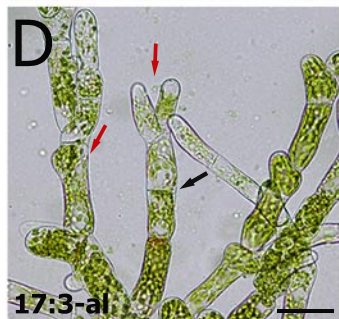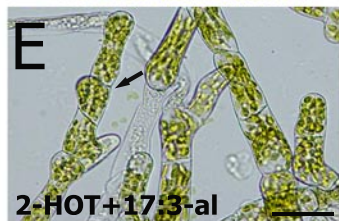

Supplement: Additional file 6: — Effect of α-DOX-derived oxylipins on chloronemal development. (A) Repartition of chloronemal cell length (in micrometers) in protonemal tissues grown on chloronemal induction conditions for 6 days. Black columns, pUBI:Ppα-DOX-3; dark gray columns, wild-type; light gray columns, Ppα-dox-2. (B) Values of average chloronemal cells length (in micrometers) measured in wild-type tissues grown for 6 days in 50 μM 17:3-al, 50 μM 2-HOT and 50 μM 2-HOT+ 50 μM 17:3-al-containing medium in comparison with control plants grown on 0.5% ethanol. Wild-type protonemal filaments showing typical phenotype after 6 days of growth on chloronemal induction conditions on 50 μM 17:3-al-containing BCD medium (D), 50 μM 2-HOT+ 50 μM 17:3-al-containing BCD medium (E), in comparison with control plants grown on 0.5% ethanol containing BCD medium (C). Black arrows in C-E indicate septa between cells. Red arrows in D indicate abnormal cell division. Asterisks for chloronemal cells length grown for 6 days in 17:3-al or HOT+ 17:3-al-containing medium (B), indicate that the values are significantly different from chloronemal cells length grown for 6 days in 0.5% ethanol, according to Kruskal–Wallis test: P <0.001. Scale bars: 20 μm. [file 12870_2015_439_MOESM6_ESM.pdf]
